# Improved electrochemical and photocatalytic activity of datura leaf extract mediate ZnO nanoparticles for the degradation of synthetic organic dyes

**DOI:** 10.1039/d6ra07680g

**Published:** 2026-10-14

**Authors:** Aslam Khan, Aneela Tahira, Khalida Parveen Mahar, Muhammad Ali Bhatti, Elmuez Dawi, Lama Saleem, Riyadh H. Alshamari, Zafar Hussain Ibupoto, Ayman Nafady

**Affiliations:** a Institute of Chemistry, Shah Abdul Latif University Khairpur Mirs Sindh Pakistan; b Institute of Chemistry, University of Sindh Jamshoro 76080 Pakistan zaffar.ibhupoto@usindh.edu.pk; c Centre for Environmental Sciences, University of Sindh Jamshoro 76080 Sindh Pakistan; d College of Humanities and Sciences, Department of Mathematics and Sciences, Ajman University P.O. Box 346 Ajman United Arab Emirates; e Chemistry Department, College of Science, King Saud University Riyadh 11451 Saudi Arabia; f Biomolecular Science, Earth and Life Science, Amsterdam University 1081 HV Amsterdam Netherlands

## Abstract

In photocatalytic and electrochemical applications, it is highly desirable to develop environmentally friendly and scalable methods for synthesizing semiconductor nanoparticles. In this study, ZnO nanoparticles were synthesized *via* a green, datura leaf extract by using hydrothermal method. A series of samples were prepared *via* using varying extract volumes of 2 mL (sample 1), 4 mL (sample 2), 6 mL (sample 3), and 8 mL (sample 4) of datura leaf extract. Structural characterization using SEM, PXRD, FTIR, and EDS confirmed the formation of uniform ZnO nanoparticles with a hexagonal wurtzite phase and dominant Zn–O bonding, with Zn and O as the principal elements. UV-visible spectroscopy revealed a progressive decrease in the optical band gap with increasing datura extract concentration, indicating improved visible-light absorption. Photocatalytic performance was evaluated for the degradation of malachite green (MG) and (MB) under natural sunlight. The sample 4 of ZnO nanoparticles exhibited superior activity, achieving 99.8% degradation of 3.8 × 10^−5^ M MG at pH 12 after using 15 mg catalyst. While the 98.3% degradation of MB in 2.81 × 10^−5^ M was observed for the sample 4. Systematic investigation of operational parameters, including catalyst dosage, dye concentration, and solution pH, confirmed their significant influence on degradation efficiency. Reactive species trapping experiments indicated that e^−^, h^+^, and ˙OH radicals play dominant roles in the photocatalytic process. Furthermore, sample 4 of ZnO demonstrated excellent reusability, maintaining high degradation efficiency over multiple cycles, highlighting its stability and practical applicability for wastewater treatment. The sample 4 of ZnO was seen highly active for the electrochemical detection of MB in 0.1 M NaOH and observed performance was witnessed through the detection range of 0.005 mM to 4 mM and a low limit of detection (LOD) was found to be about 0.0005 mM. The enhanced photocatalytic and electrochemical performance were attributed to the surface modification, band gap tuning, and improved charge carrier dynamics of ZnO. This study establishes a cost-effective and sustainable platform for designing high-performance ZnO-based photocatalysts for environmental remediation applications.

## Abstract 2

In photocatalytic and electrochemical applications, it is highly desirable to develop environmentally friendly and scalable methods for synthesizing semiconductor nanoparticles.

## Graphical abstract

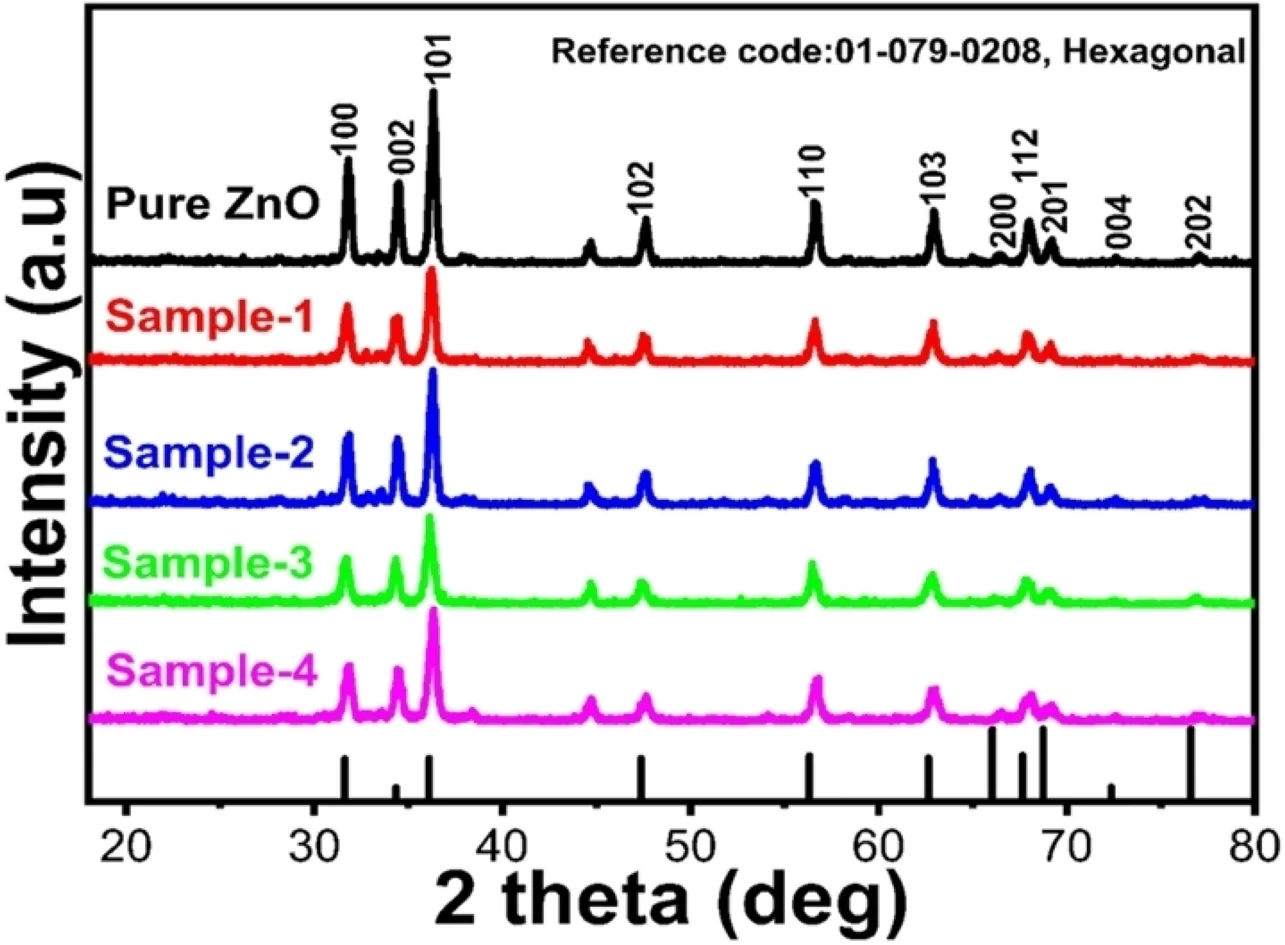

## 1 Introduction

Nanoparticles of metal oxides have broad applications across magnetism, nonlinear optics, environmental science, medicine, electronics, pharmacy, antimicrobial action, textiles, consumer goods, and heavy industry, showcasing remarkable properties. The enhanced electronic, optical, conductive, magnetic, and chemical characteristics of metal oxides, including AgO, NiO, Al_2_O_3_, CuO, MgO, TiO_2_, and ZnO, have been attributed to small particle size and a high surface-to-volume ratio.^1–7^ Zinc oxide (ZnO) stands out as a notable nanomaterial, extensively studied relative to iron-based oxides, and possesses intrinsic capabilities to modulate morphology through variation of precursors and synthesis technologies. With a wide band gap of 3.37 eV and an exciton binding energy of 60 meV, ZnO enables broad applicability in chemical sensing, photocatalysis, and photovoltaic devices owing to its distinctive electrical, optical, and photocatalytic properties.^8,9^ ZnO is also employed in cell labeling, drug delivery, biosensing, nanomedicine, gene transfer, and activities such as antibacterial, antifungal, antidiabetic, anticancer, and antilarvicidal applications.^10,11^ The synthesis of ZnO nanoparticles has employed methods such as laser ablation,^12^ solvothermal,^13^ sol–gel,^14^ and chemical reduction,^15^ each associated with challenging reaction conditions, including toxic reagents, high temperature and pressure, and inert atmospheres such as helium or laser radiation. In response, greener approaches using non-toxic, low-cost reagents have gained prominence; phytochemical-mediated preparation routes are increasingly regarded as promising and viable alternatives for high-performance nanostructured materials.^16,17^ The reducing, structure-directing, chelating, and stabilizing properties of phytochemicals from plant sources facilitate tuning of ZnO nanostructures' structural and chemical properties, with flavonoids and phenolic compounds markedly influencing morphology and catalytic and antimicrobial activity. The antibacterial and photocatalytic efficiency of ZnO nanoparticles is highly dependent on the properties achieved *via* green synthesis routes.^18,19^ Phytochemical-based green synthesis contributes to the preparation of surface modified ZnO nanoparticles, as phytochemicals provide stabilizing and capping interactions that chelate zinc ions during growth; subsequent size controlled ZnO nanoparticles can be obtained. Stabilization and capping by phytochemicals also mitigate agglomeration, limiting the formation of large aggregates. Consequently, ZnO has been produced using various plant extracts.^20,21^ Moreover, industrial effluents are discharged directly or indirectly into water bodies, causing pollution. The wastewater from industries contains various synthetic dyes such as methylene blue (MB) and malachite green (MG) that exhibit chronic environmental hazards due to their chemical stability and toxicity to humans and aquatic ecosystems.^22–25^ Malachite green (MG) is among these synthetic dyes, belonging to the cationic class,^26,27^ and is extensively used in medicine,^28^ textiles,^29^ and food production.^30^ Exposure to MG at sub-ppm concentrations (<1 ppm) can induce cellular damage, genetic disorders, and cancer.^31^ Likewise, the use of MB is also widely spread in different industries such as cosmetic, textile, paper and leather and it has been accompanied into the water resources and environment *via* direct or indirect discard.^32^ Like other synthetic dyes, it is one of the stable and highly toxic to human health, hence MB exposure causes hypertension, anxiety, irritation, vomiting, nausea, abdominal pain, dizziness and headache.^33^ Therefore, it is essential to design new approaches with high sensitivity for the detection of MB.

Consequently, the removal of MG from aquatic systems is imperative to mitigate environmental and health risks. Traditional remediation methods, including adsorption,^34–36^ sonochemical degradation,^37,38^ membrane filtration,^39^ and biological treatment,^40–42^ have been employed for MB and MG removal but are often inefficient, costly, and may generate secondary pollutants.^43^ Given these limitations, advanced oxidation processes (AOPs) leveraging photocatalytic materials have attracted considerable attention for dye decomposition,^43–45^ with the photocatalytic mechanism relying on *in situ* generation of reactive oxidative species capable of mineralizing a broad range of organics to CO_2_ and H_2_O.^46^ Also, the electrochemical method has received significant attention for the detection of toxic dyes such as MB owing its excellent advantageous features including highly sensitivity, rapid, low cost and simple to use.^47–49^ Datura stramonium (jimsonweed or thorn apple), a plant widely distributed across tropical and subtropical regions, is rich in phytochemicals such as carbohydrates, flavonoids, glycosides, amino acids, phenolic compounds, and tannins. These bioactive constituents serve as multifunctional agents, acting as reducing, stabilizing, and capping species during nanoparticle synthesis. In particular, carbohydrates and flavonoids contribute to reduction and stabilization processes, while phenolic compounds enhance surface passivation and structural control. The role of these phytochemicals towards the surface and optical modification of ZnO nanoparticles for the efficient photocatalytic removal of MG and MB from aqueous systems have not been extensively explored. Also, the role of datura leaves extract for the preparation of ZnO nanoparticles towards sensitive electrochemical detection of MB has not been demonstrated previously.

In the present work, datura-derived phytochemicals are employed to regulate the nucleation and growth of ZnO nanoparticles, leading to the formation of uniform and surface-engineered nanoparticles. A generalized growth mechanism is proposed for the photocatalytic degradation pathway of MG/MB.

## 2 Experimental section

### 2.1. Materials utilization

Datura leaves were collected from the deserted land of the University of Sindh Jamshoro, Sindh, Pakistan. Zinc acetate dihydrate (98%), potassium hydroxide (85%), hydrochloric acid (37%), acetone (99.5%), ethanol (99.5%), methylene blue (82%), aqueous ammonia (33%), sodium borohydride (98%), silver nitrate (99%), di-sodium ethylenediaminetetraacetate (99.4%), isopropyl alcohol (70%) and sodium hydroxide (97%) were the chemical reagents used in the presented study. Sigma-Aldrich, Karachi, Sindh, Pakistan was the chemical supplier for these chemical reagents. Deionized water was employed throughout the research. All photocatalytic experiments were conducted in aqueous solution.

### 2.2. Preparation of datura leaves assisted ZnO nanoparticles

The fresh leaves of datura were picked, and they were washed with the deionized water. Then, they were chipped into small pieces, and a mass of 100 g were added into 300 mL of the deionized water. The beaker was covered with aluminum sheet and placed on the hot plate at 60 °C with constant magnetic stirring for 1 hour. Then, the filtration was carried out using ordinary filter paper and filtrate was obtained which was labeled as datura leaves extract. A 0.1 M zinc acetate dihydrate was taken into four different beakers containing 5 mL of 33% aqueous ammonia solution. Then, 2 mL (sample 1), 4 mL (sample 2), 6 mL (sample 3) and 8 mL (sample 4) of datura leaves extract was added in these four beakers and total volume of growth solution was made up to 100 mL by adding deionized water. The aluminum sheet was used to seal these four beakers containing growth precursors and left in furnace at 95 °C for 5 hours. The datura leaves extract induced the different phytochemicals during the growth process as reducing, capping, stabilizing and structure directing agents to control the size and shape of as produced nanoparticles. However, the purpose of using ammonia solution was to accelerate the growth kinetics through the provision of hydroxide ions for producing the significant amount of zinc hydroxide during the hydrothermal process. With the completion of growth process, the product was collected on filter paper. Using same method without datura leaves extract, zinc hydroxide-based nanoparticles were obtained. Then, this prepared zinc hydroxide-based nanoparticles were placed in an electric furnace at 300 °C in air for 3 hours in order to get high purity ZnO material and consequently the presented ZnO materials were ready for the structural and photocatalytic experiments. A brief and stepwise synthesis of datura mediated ZnO nanoparticles is shown in Scheme 1. The phytochemicals after calcination could not be seen in the material because the pyrolysis usually becomes highly active at 300 °C, the most of phytochemical undergoes to severe oxidation, hydrolysis and cleavage into lighter volatile gases and leaving behind carbon residual particles such as char. As in the presented study, a small volume of extract was used and the contribution phytochemicals to char at 300 °C would be very small, hence, there could be negligible role of char to photocatalysis compares to the presence of bulk amount of ZnO during photocatalytic experiments. The XRD measurements were performed on a D8 Advance diffractometer (Bruker, Germany) for observing the phase of different ZnO nanoparticles. Morphological features of ZnO nanoparticles were studied on scanning electron microscopy (SEM) using a voltage of 10 kV. The elemental analysis was done through energy dispersive spectroscopy connected to SEM. The optical properties of datura mediated ZnO and photocatalytic experiments were carried out on a 1800 PC UV-vis spectroscope (Mapada, China) having a deuterium lamp. The Zn–O chemical bonding characteristics were analyzed using Fourier transform infrared (FTIR) spectroscopy.

**Scheme 1:**
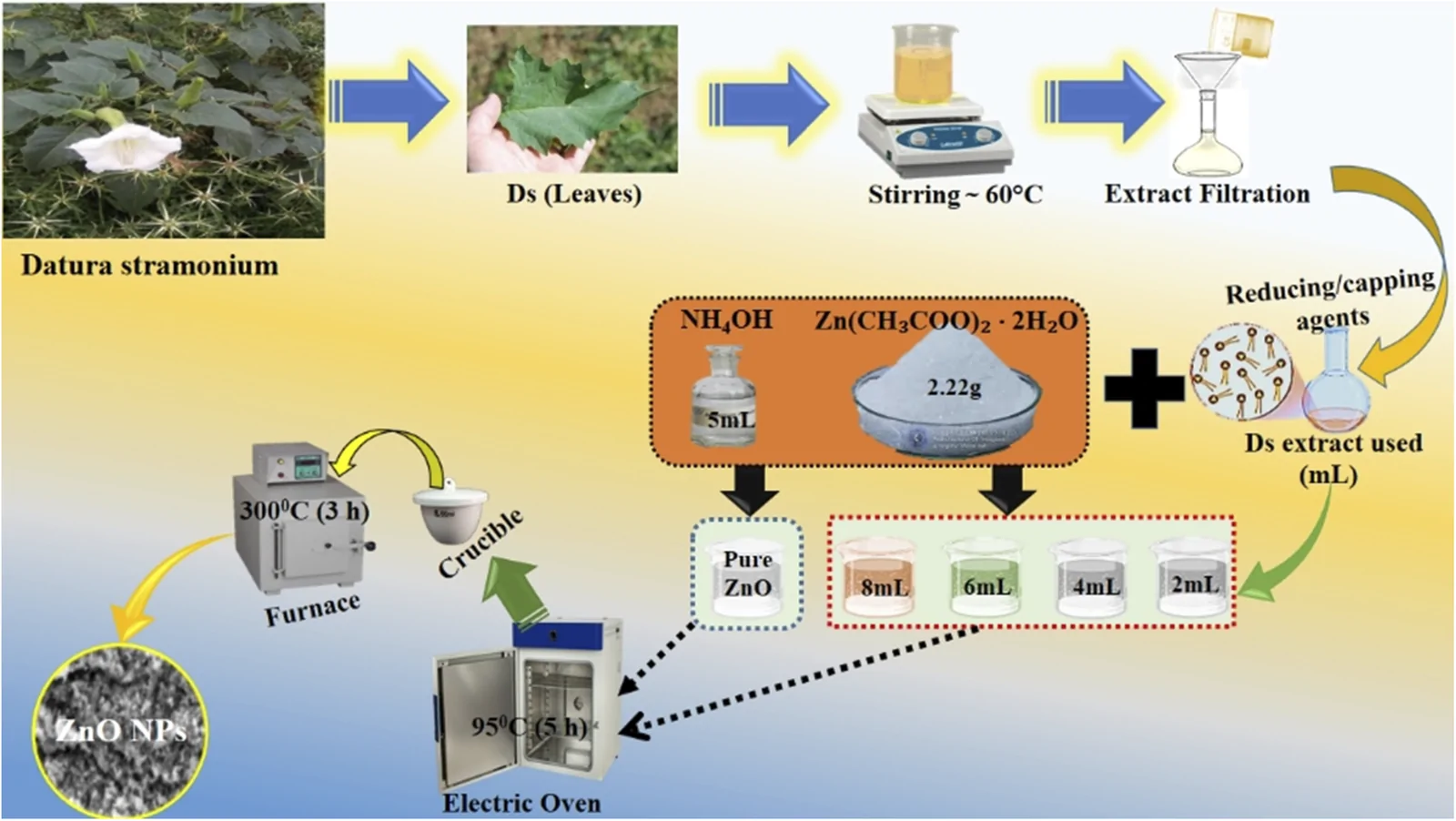
Stepwise synthesis of datura mediated ZnO nanoparticles.

### 2.3. Use of datura leaves extract mediated ZnO nanoparticles for the degradation of dyes

The role of datura leaves extract was studied for the modification of surface and optical properties of ZnO and their attribution to improved solar light irradiation photocatalytic degradation of malachite green (MG) and methylene blue (MB). The low and high MG concentrations such as 3.8 × 10^−5^ M and 8.2 × 10^−5^ M were prepared in deionized water. First datura mediated ZnO (sample 1 to sample 4) and pure ZnO with a catalyst dose of 10 mg were studied in 8.2 × 10^−5^ M of MG. MB concentration was 2.81 × 10^−5^ M prepared and contained 15 mg catalyst dose. Before photocatalytic measurements, the covered dye solution containing 10 mg of each catalyst and for 30 min was stirred in ultrasonic bath to ensure the adsorption–desorption equilibrium in dark. For 150 min, dye solution was irradiation with sunlight, and the photocatalytic experiments were performed after each 25 min. The degradation absorption spectra were monitored by UV-visible spectrometer having quartz squire cuvette with a 10 mm (1 cm) optical path length taking approximately 2 mL of degraded dye solution for each reaction. The use of 3.8 × 10^−5^ M of MG was considered for the evaluation of effect of catalyst dose and pH. The pH adjustment in dye solution was done with 2 M HCl and 2 M NaOH. There has been minor variation in the dye color for pH 12 after the use of very little content of concentrated HCl and NaOH for adjusting pH. Therefore, dye degradation under the neutral pH 7 was also examined. The scavenger measurements were done for trapping reactive species using 20 ppm of di-sodium ethylenediamine tetraacetate, isopropyl alcohol (IPA), sodium borohydride, and silver nitrate in 3.8 × 10^−5^ M dye solution. The cycling stability was studied using 15 mg catalyst dose of sample 4 of datura mediated ZnO in 3.8 × 10^−5^ M. The dye degradation efficiency was estimated using under given relation:

1 % Dye removal = (*C*_0_ − *C*_*t*_)/*C*_0_ × 100 = (*A*_0_ − *A*_*t*_)/*A*_0_ × 100

Herein, *C*_0_ and *C*_*t*_ represent MG dye concentrations before the start of degradation and after degradation at a specific time, respectively, and *A*_0_ and *A*_t_ indicate the corresponding absorbance values of MG before the irradiation of light and after the irradiation of light respectively.

The pseudo first order equation as given below was followed to examine the dye degradation kinetics.

2 (*C*_*t*_/*C*_0_) = exp(−*kt*)

Whereas *k* as rate constant (min^−1^), *C*_*t*_ is dye concentration after dye degradation, *C*_0_ dye concentration before the degradation.

Electrochemical measurements were done on the different modified electrodes using pure ZnO, and datura leaves extract mediated ZnO (sample 1 to sample 4) in 0.1 M NaOH. Cyclic voltammetry (CV), chronoamperometry and electrochemical impedance spectroscopy (EIS) measurements were carried out on a potentiostat/galvanostat (Gamry Instruments Inc.) using a three-electrode cell configuration with Ag/AgCl electrode as a reference electrode and platinum wire as a counter electrode and working electrode was based on glassy carbon electrode (GCE). The cleaning of GCE was performed by using alumina paste and carbon paper followed by washing with deionized water and ethanol. The drying of GCE was carried out at room temperature. The material ink was received by the mixing 3 mg of each ZnO material in 2 mL of deionized water, 50 µL of 5% nafion and 0.5 mM of isopropanol. The depositing of material ink on the GCE was done *via* drop cast method and subsequently the material adherence to working electrode was carried out by drying modified GCE with air blow. The CV encompassing a potential range spinning −0.8 to +0.8 V and employing scan rate between 10 mV s^−1^ to 2010 mV s^−1^, and EIS experiments were employed at 100 000 kHz to 0.1 Hz and using 5 mV and oxidation potential of MB as biasing potential in 0.1 M NAOH. The electrochemical analysis was for the dye detection was carried out in 0.1 M NaOH as supporting electrolyte. Each dye with stock concertation of 10 mM was prepared in 0.1 M NaOH. The stock concentrations were further utilized for obtaining the low concentrated dye solutions.

## 3 Results and discussion

### 3.1. Structural, morphological, composition and optical analysis of datura mediated ZnO

The structural composition and crystal arrays of datura mediated ZnO (sample 1–sample 4) and pure ZnO were using XRD^47^ as seen in Fig. 1. The measured diffraction patterns were plotted against the two-theta angle and various reflection peaks were noticed at 31.769°, 34.421°, 36.252°, 47.538°, 56.602°, 62.862°, 66.378°, 67.961°, 69.098°, 72.560°, and 76.953°, correlated to XRD patterns (100), (002), (101), (102), (110), (103), (200), (112), (201), (004), and (202), respectively. The obtained XRD results suggested the presence of hexagonal wurtzite phase for ZnO and these patterns were in good agreement with the standard JCPDS card no: 01-079-0208. There were not any patterns related to any other phase or substance in the datura mediated ZnO nanoparticles. Compared to pure ZnO, the relative peak intensity of datura mediated ZnO was decreasing with successive addition of datura leaves extract and obvious decrease in the peak intensity could be seen for the sample 4. Because of the higher content of phytochemicals in case of sample 4 that has changed the growth kinetics. The estimation of crystallite size for pure ZnO and datura leaves extract mediated ZnO (sample 1 to sample 4) was done as shown Table S1. The comparison of crystallite size indicated that there was a variable crystallize size for the materials because crystallite size depends on different factors such as synthetic conditions, and in our case each material was produced at different conditions, hence we observed the varied crystallite size. The decrease in the intensity of XRD reflections could be attributed to partial amorphous surface layers modified by plant phytochemicals.

**Fig. 1:**
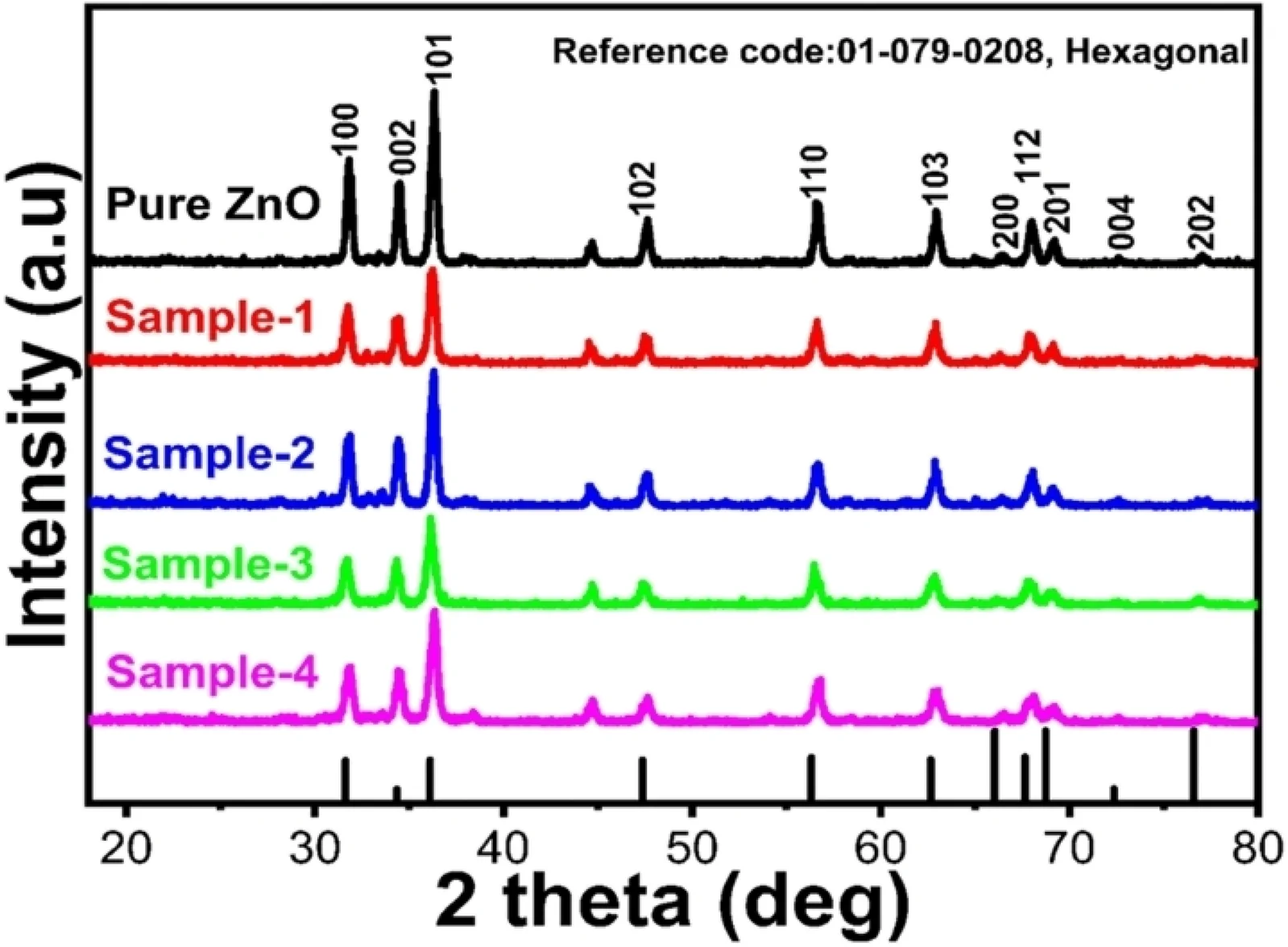
Powder XRD diffraction patterns of pure ZnO, datura mediated ZnO (sample 1–sample 4).

The SEM studies were performed on pure ZnO and datura leaves extract mediated ZnO (sample 1 to sample 4) for investigating the shape as seen in Fig. 2. The comparative study was carried out for the datura mediated ZnO samples with pure ZnO. The SEM observations suggested the fine particle like surface morphology of all the ZnO materials shown in Fig. 2a–e. The structure of presented ZnO was heterogeneous in terms of size, however the surface morphology was uniform throughout the samples.

**Fig. 2:**
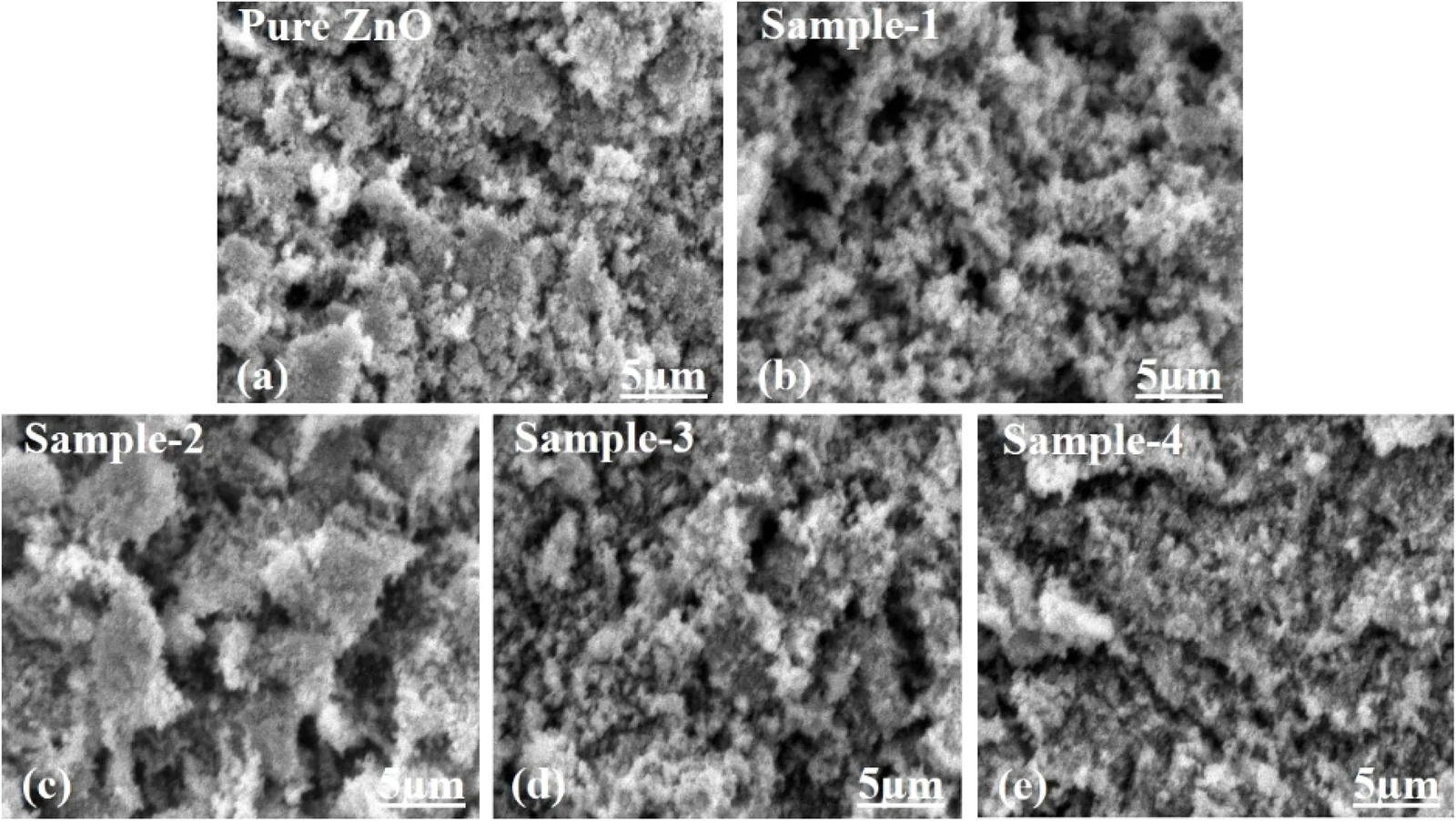
SEM micrographs (a) pure ZnO, (b–e) datura mediated ZnO (sample 1–sample 4).

The heterogeneous size could be attributed to the relative increase in the amount of datura leaves extract which carried different amounts of phytochemicals, thus these naturally reducing and capping agents could later the reaction rate during the growth process. The influence of phytochemicals on the growth kinetics of ZnO was generalized as shown in Scheme 2. The phytochemicals might produce the complex with zinc ions and thereby reduce some of the zinc ions, later under thermal treatment, transformed into ZnO.

**Scheme 2:**
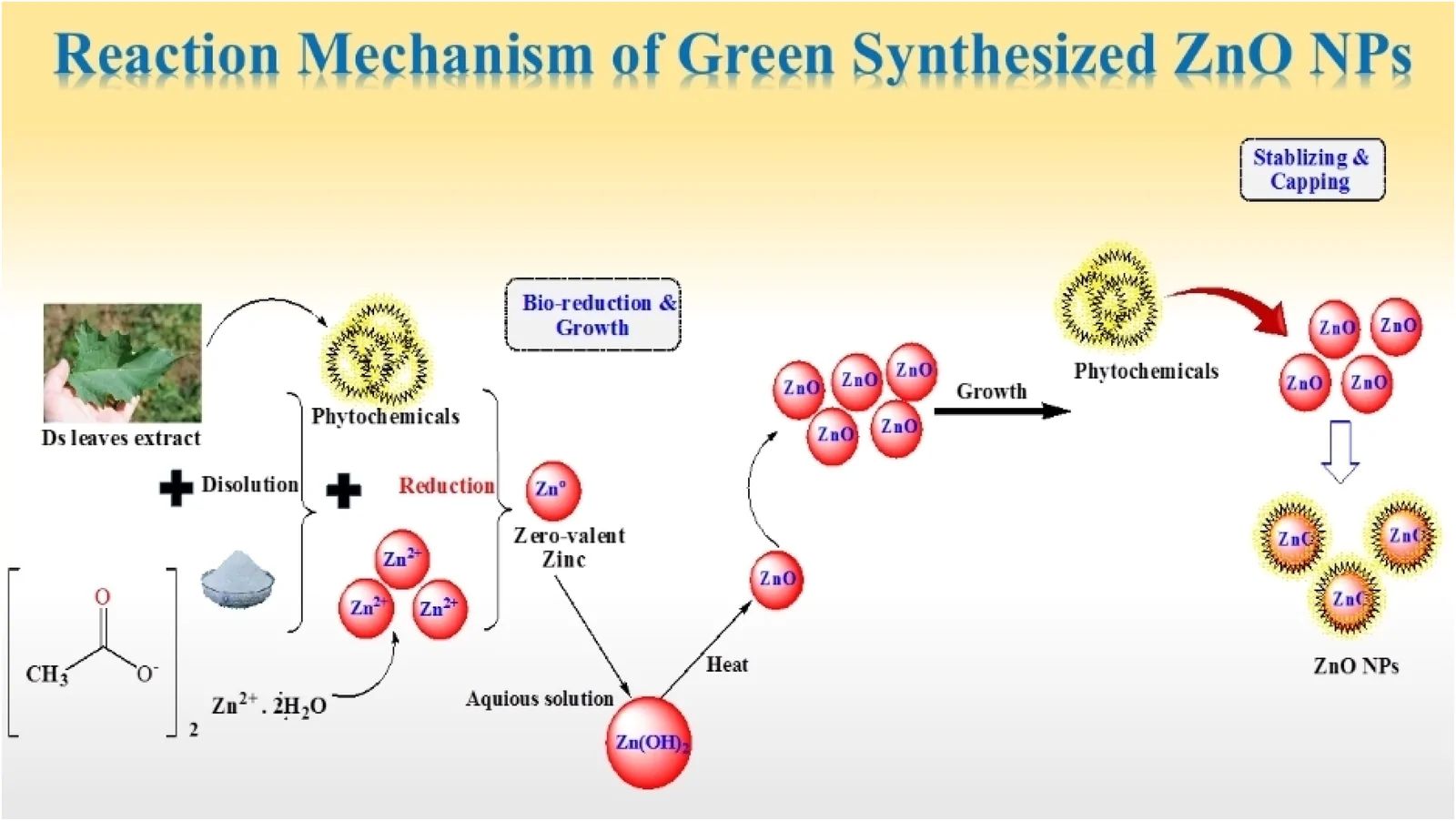
Proposed growth mechanism of ZnO under the influence of phytochemicals from datura leaves extract.

The elemental analysis was performed on the pure ZnO and datura mediated ZnO (sample 1–sample 4) using energy dispersive spectroscopy (EDS). Each ZnO material was carrying the Zn and O as the main elements as shown in Fig. 3a–e. The elemental analysis of datura mediated ZnO and pure ZnO has verified the chemical composition.

**Fig. 3:**
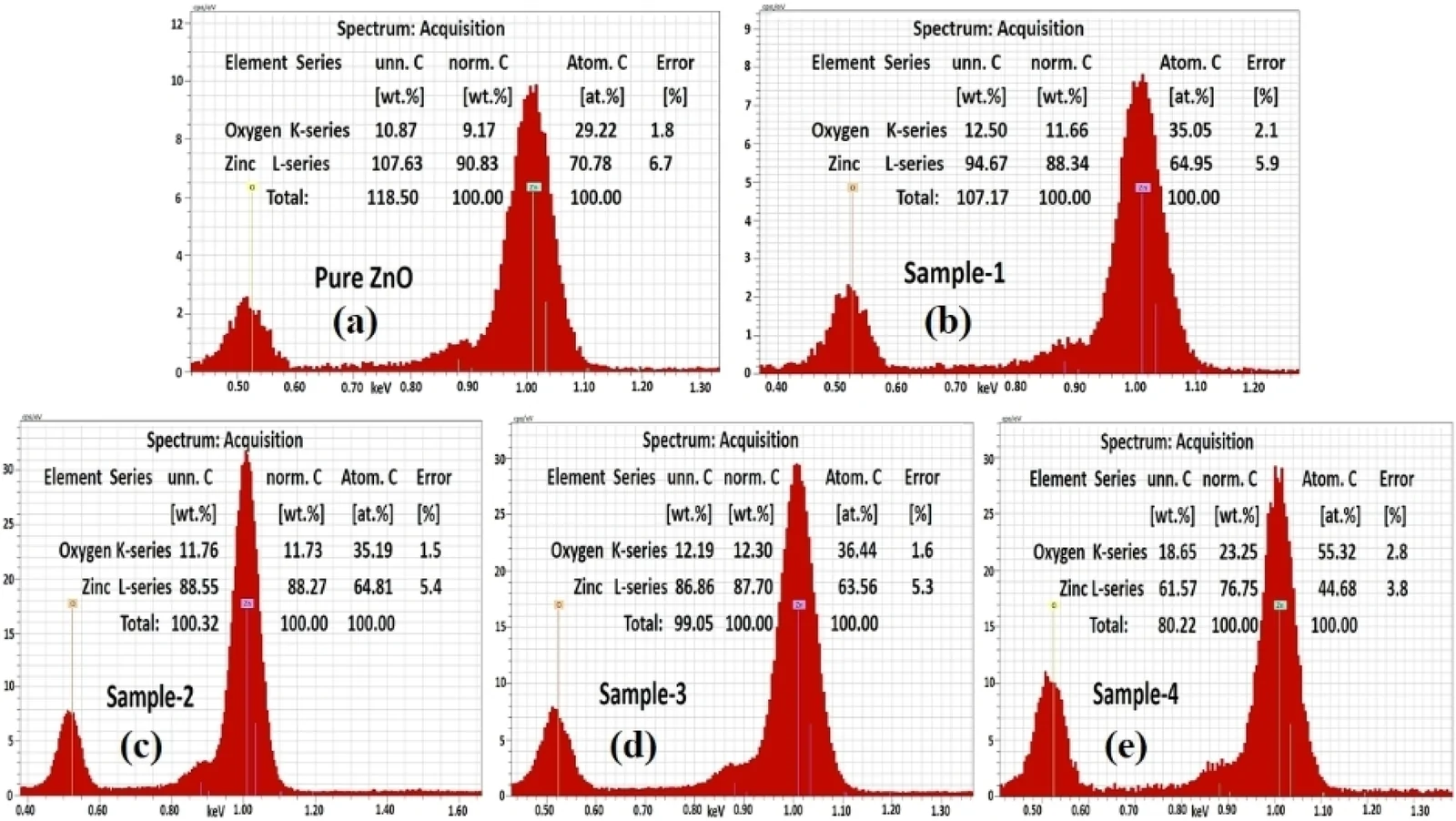
EDS spectra (a) pure ZnO, (b–e) datura mediated ZnO (sample 1–sample 4).

The successful formation of ZnO using datura leaves extract was further verified *via* FTIR experiments. The appearance of IR bands for the Zn–O can confirm the formation of ZnO.^48^ Additionally, the presence of other functional groups could be viewed and identified using FTIR analysis as shown in Fig. 4. The FTIR spectra carried the Zn–O, –C–OH, –C≡C–, and –OH (water) bonds and these could be verified through Fig. 4. The ZnO shows the typical IR peak in the region of 400 to 700 cm^−1^.^49,50^ The appearance of 513 cm^−1^ and 617 cm^−1^ IR bands has confirmed the ZnO presence in each sample and supported the observations described about the ZnO formation by XRD analysis. The occurrence of C–OH bending and C–OH stretching vibrations were seen at 1028 cm^−1^ and 1384 cm^−1^ respectively. The appearance of IR band at 3495 cm^−1^, indicating the –OH band stretching frequency because of adsorbed water molecules. A IR frequency at 1636 cm^−1^ attributed to asymmetric C=O bond vibrations. It was also viewed that the datura mediated ZnO has carried out the slight shift in the respective IR bands and it could be indexed to the particle size, surface morphology and the interaction of phytochemicals with ZnO. FTIR analysis has expressed the formation of ZnO, however additional IR bands could be attributed to certain impurities and the occurrence of phytochemicals.

**Fig. 4:**
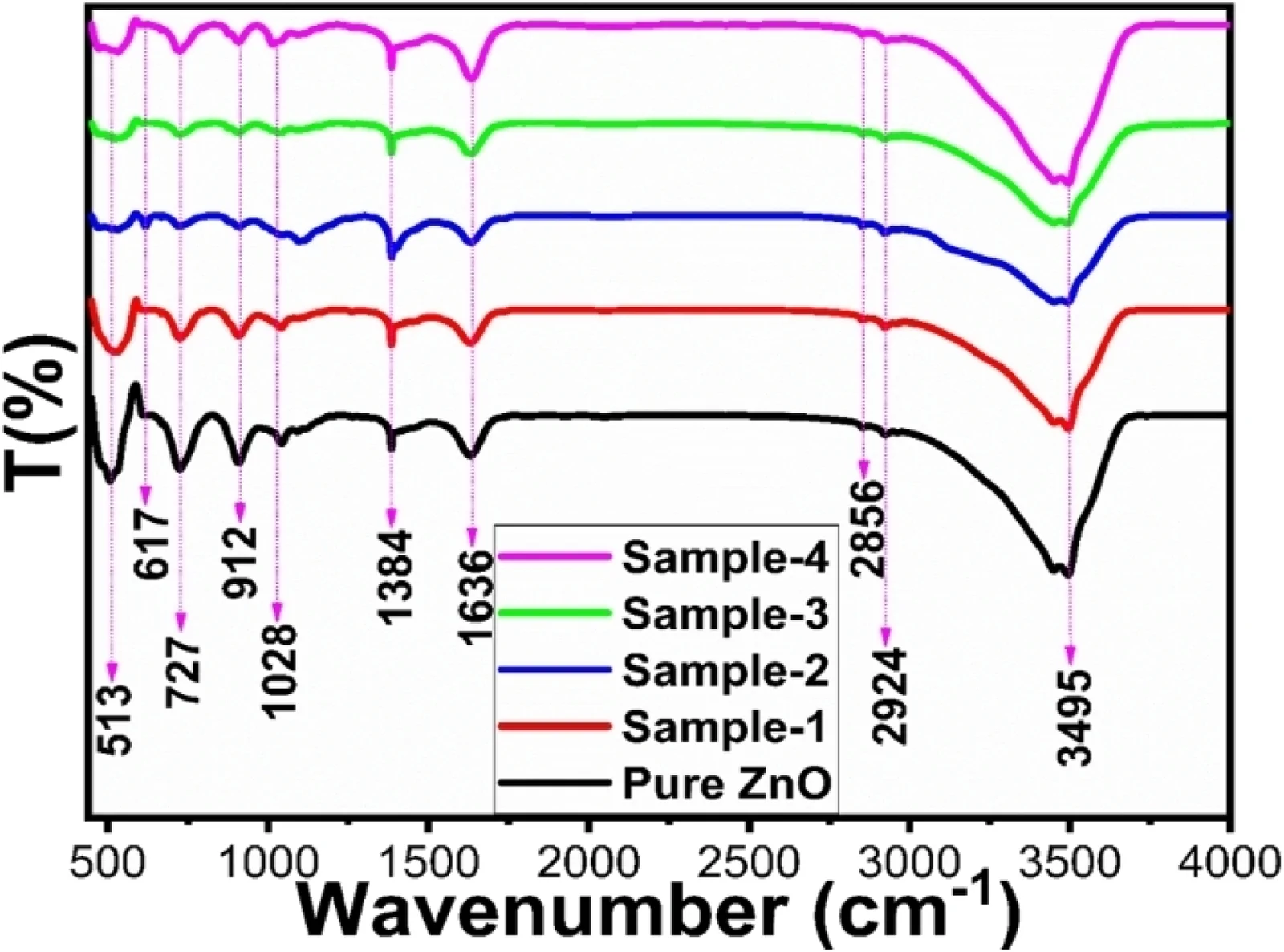
FTIR spectra of pure ZnO, datura mediated ZnO (sample 1–sample 4).

The influence of datura leaves extract on band structure of ZnO was also examined by UV-visible diffuse reflectance. The diffuse reflectance spectra of pure ZnO and datura mediated ZnO (sample 4 to sample 1) were measured as shown in Fig. 5a. Each sample of ZnO was characterized with the strong absorption edge in the region of above 380 nm which is the typical characteristic region of ZnO, revealing the formation of ZnO.^51^ The observed red shift has been contributed to the defects induced by the phytochemicals, surface modification and the possible slight attribution from the carbon residual species like char. The optical band gap of as produced ZnO materials was calculated by plotting (*F*(*R*)*hν*)1/2 *vs.* photon energy (*hν*). Herein, (*F*(*R*)) is Kubelka–Munk Function and *hν* is photon energy. The linear plot was used to estimate the band gap as shown in Fig. 5b. The pure ZnO, sample 1–sample 4 exhibited the optical band gap value as 3.22, 3.13, 3.05, 2.98 and 2.90 respectively using the extrapolation of linear part of respectively as shown in inset of Fig. 5b. The reduction in the band gap could be contributed from the surface morphology, defects and used synthetic route.^52,53^ Therefore, the sample 4 of ZnO resulted the lowest optical band gap.

**Fig. 5:**
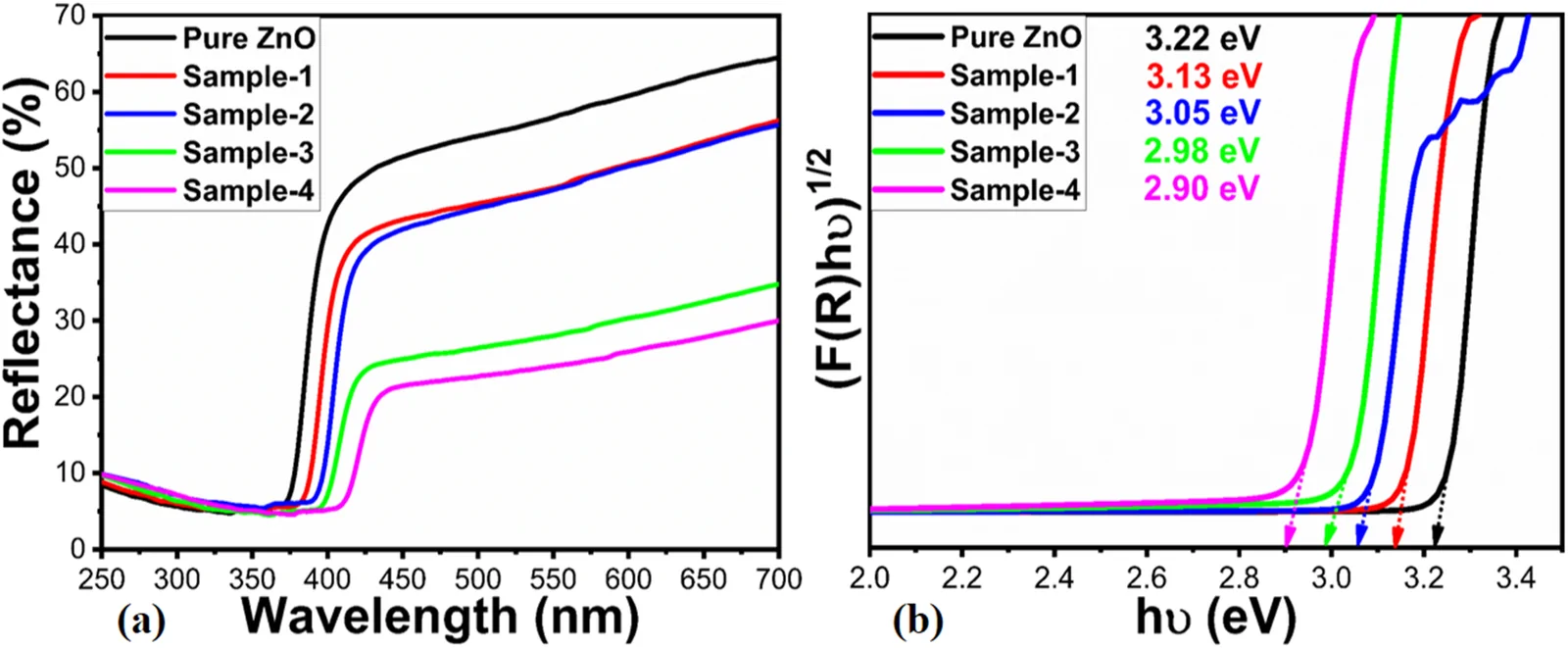
UV-visible reflectance spectra (a) pure ZnO and datura mediated ZnO (sample 1–sample 4) (b) showing the Tauc's plots with estimated optical band gap.

### 3.2. Photocatalytic performance of datura leaves extract mediated ZnO nanoparticles

To illustrate the functionality of datura leaves, extract mediated ZnO (sample 1 to sample 4), the photocatalytic activity was studied against the decomposition of malachite green (MG) using sunlight. The role of datura leaves extracts in enhancing the photocatalytic performance of ZnO was compared with the ZnO synthesized without datura leaves extract (pure ZnO). The time dependent UV-visible absorption spectra were obtained between 25 min to 150 min. First, the performance of pure ZnO was evaluated for the degradation of MG using 10 mg of catalytic dose and dye concentration of 8.2 × 10^−5^ M. The adsorption–desorption equilibrium was achieved in the dark at constant stirring for 30 min. Then, the dye solution containing pure ZnO was exposed to natural sunlight during the month of Jun 2024 at 11:30 am to 3:30 pm. All the photocatalytic measurements were done during that time. Then, the effectiveness of pure ZnO towards the degradation of MG was analyzed by taking the 1 mL of dye solution with each interval of 30 min. The measured absorption spectra for the different intervals of MG are shown in Fig. 6a. The estimation of dye degradation could be seen through the relative decrease in the absorbance value. It was observed that the difference between consecutive absorbance values is very small, indicating the inefficiency of pure ZnO towards the catalytic degradation of MG. The pseudo first order equation was employed to describe kinetics of degradation process, and the linear plots were obtained as shown in Fig. 6b. The observations indicted the low-rate constant value of 1.99 × 10^−3^ M as given in Table 1. Also, the degradation rate was examined as shown in Fig. 6c, and the relative degradation rate with different intervals of time is sluggish. The dye removal percentage of 25% using pure ZnO was also estimated as given in Fig. 6d. The limiting activity of pure ZnO could be attributed to several shortcomings such as wide band gap, low surface catalytic sites, and the fast recombination rate photogenerated charges. Therefore, despite pure ZnO possessing photocatalytic activity, it is very limited; thus, pure ZnO is not a suitable photocatalytic material for real wastewater applications. Hence, structural and optical disorder can lead to improved photocatalytic activity of ZnO to address real-world applications. The datura leaves extract has brought significant variability in the structure, size, shape and optical properties of ZnO, thereby can show efficient photocatalytic activity against dyes.

**Fig. 6:**
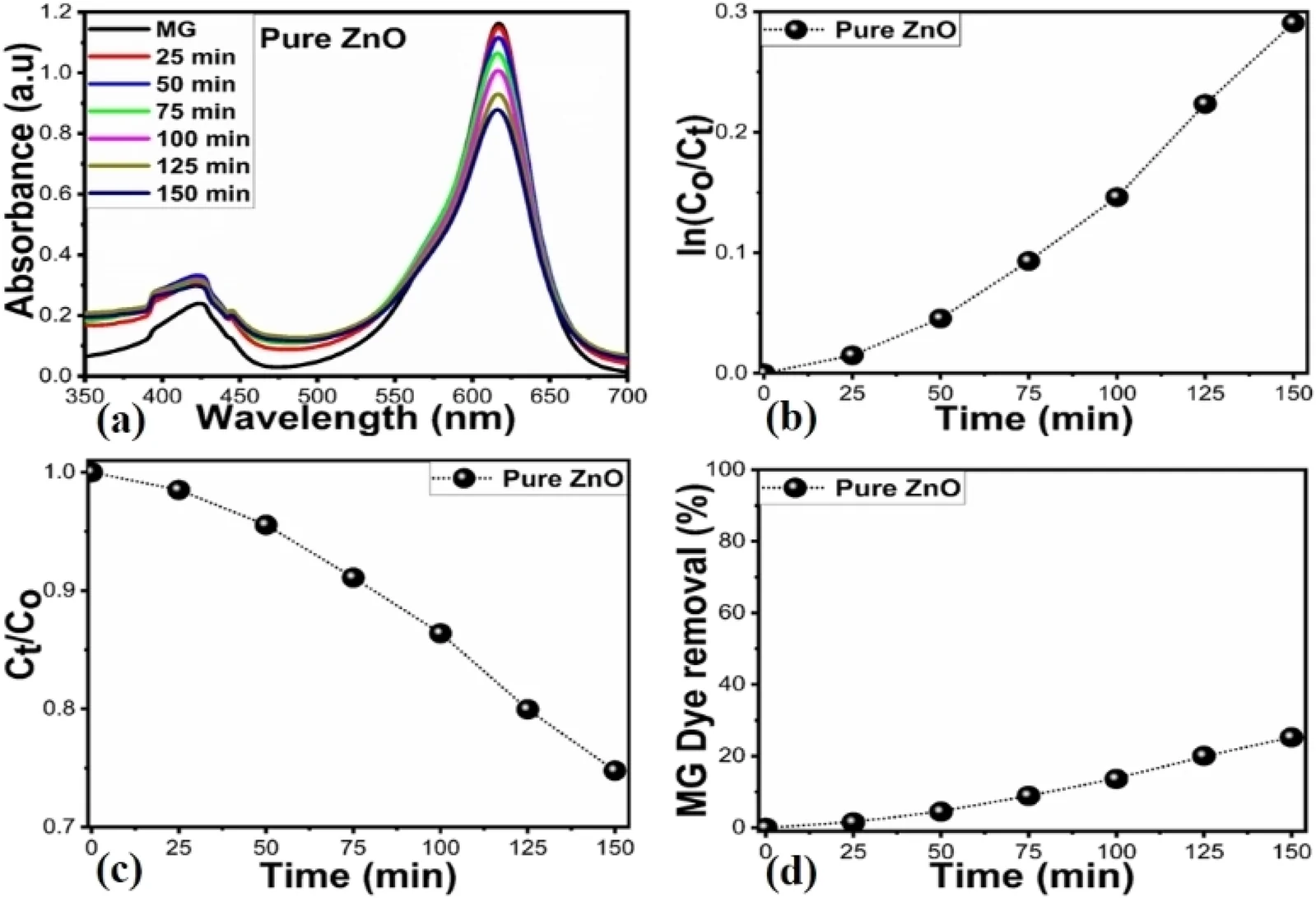
(a) UV-visible absorption spectra of MG in the presence of 10 mg catalyst dose pure ZnO in dye 8.2 × 10^−5^ M under the irradiation of natural of sunlight, (b) pseudo first kinetic plot, (c) degradation rate, (d) dye removal %.

Performance observations about pure ZnO and datura mediated ZnO

**Table:** 

Samples (ID)	Amount of dye (MG)	Time (min)	Degradation (%)	Rate constant (*K*, min^−1^)
Pure ZnO (10 mg)	8.2 × 10^−5^ M	150	25.2	1.99 × 10^−3^
Sample-1 (10 mg)			37.3	3.30 × 10^−3^
Sample-2 (10 mg)			56.6	5.50 × 10^−3^
Sample-3 (10 mg)			69.7	8.07 × 10^−3^
Sample-4 (10 mg)			79.8	1.04 × 10^−2^
Sample-4 (5 mg)	3.8 × 10^−5^ M	60 min	83.2	2.72 × 10^−2^
Sample-4 (10 mg)			88.7	3.32 × 10^−2^
Sample-4 (15 mg)			96.9	5.15 × 10^−2^

**Table:** 

Sample (ID)	pH (value)	Time (min)	Degradation (%)	Rate constant (*K*, min^−1^)
Sample-4 (15 mg)	pH-3	50	65.0	2.20 × 10^−2^
	pH-6	40	88.7	5.12 × 10^−2^
	pH-9	20	97.0	1.73 × 10^−1^
	pH-12	10	99.8	5.82 × 10^−1^

The datura leaves extract mediated ZnO (sample 1 to sample 4) with a catalyst dose of 10 mg was added in 8.2 × 10^−5^ M at the exposure of sunlight for the period of 150 min. The corresponding absorption spectra can be seen in Fig. 7a–d. A significant decreases in the absorbance value for MG was observed datura-derived ZnO than pure ZnO, suggesting the dynamic role of phytochemicals of datura leaves extract *via* modifying the structure, shape and optical properties of ZnO. The sample 4 of ZnO prepared with 8 mL of datura leaves extract has shown significant enhancement in the degradation of MG compared to sample 3, sample 2 and sample 1 as shown in Fig. 7a–d.

**Fig. 7:**
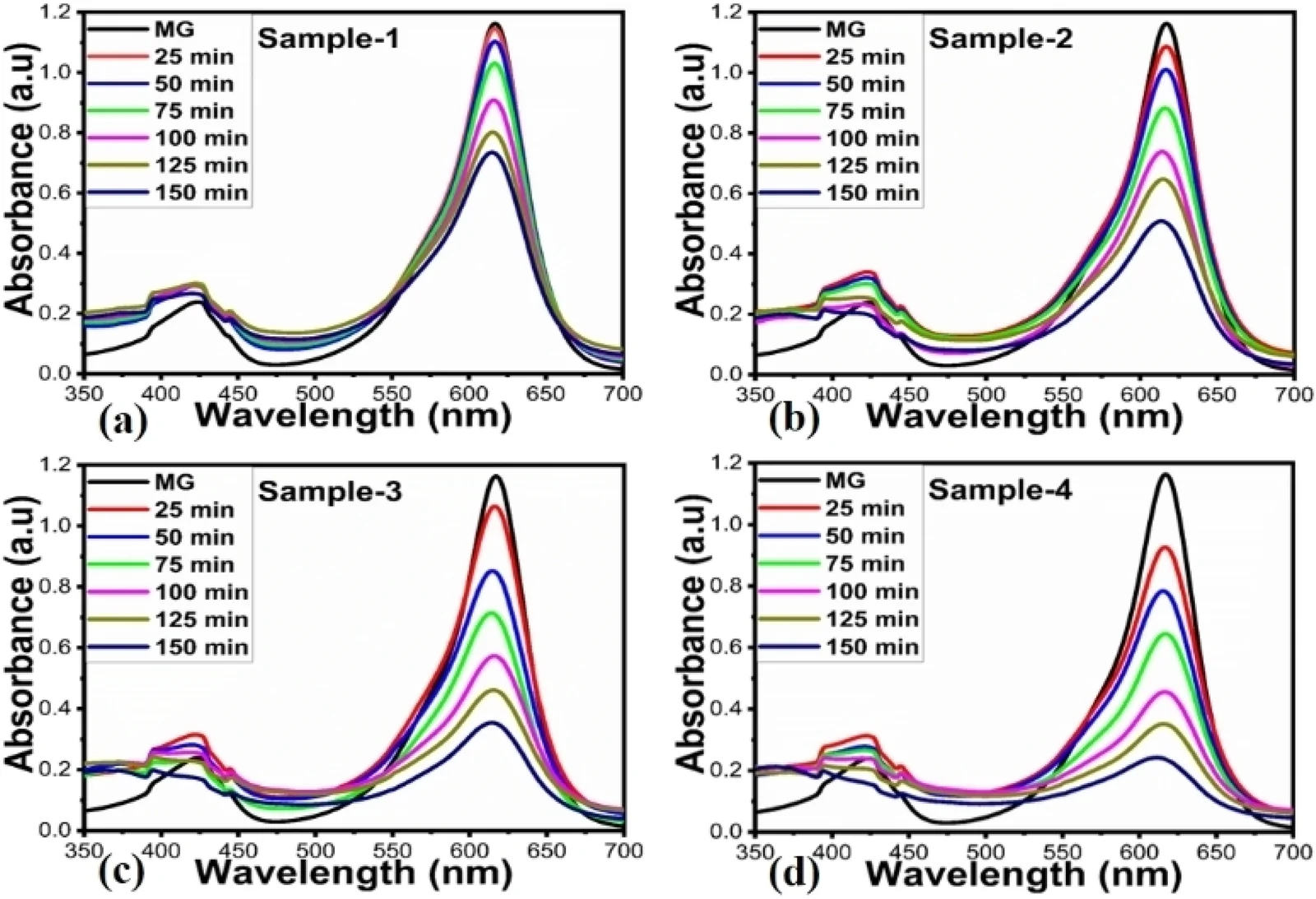
(a–d) UV-visible absorption spectra of MG in the presence of 10 mg catalyst dose datura mediated ZnO (sample 1 to sample 4) in dye 8.2 × 10^−5^ M under the irradiation of natural of sunlight.

Again, pseudo first order equation was used to investigate the dye degradation kinetics of MG using modified ZnO (sample 1 to sample 4) as shown in Fig. 8a. Its linear plots suggested that sample 4 resulted in a higher rate constant value compared to sample 3, sample 2 and sample 1 of ZnO as given in Table 1. The dye degradation was examined, and the corresponding rate is shown in Fig. 8b. It was seen that MG was degraded at a higher rate in the presence of sample 4 than sample 3, sample 2 and sample 1 of modified ZnO using datura leaves extract. The dye removal % was also monitored and the corresponding removal % is shown in Fig. 8c. It was observed that sample 2, sample 2 and sample 1 have relatively lower dye removal % than sample 4 of modified ZnO. These four modified ZnO samples prepared with various amounts of datura leaves indicate that the datura leaves extract content play a vital role in elevating the photocatalytic performance of ZnO. The 8 mL of leaves extract contained the largest number of phytochemicals which significantly altered the structure, size, uniformity and optical characteristics of ZnO; hence sample 4 demonstrated excellent photocatalytic activity against MG under the illumination of natural sunlight. This is the reason sample 4 was further used to evaluate the photocatalytic parameters such as initial dye concentration, effect of pH of dye solution, catalyst dose and cycling stability.

**Fig. 8:**
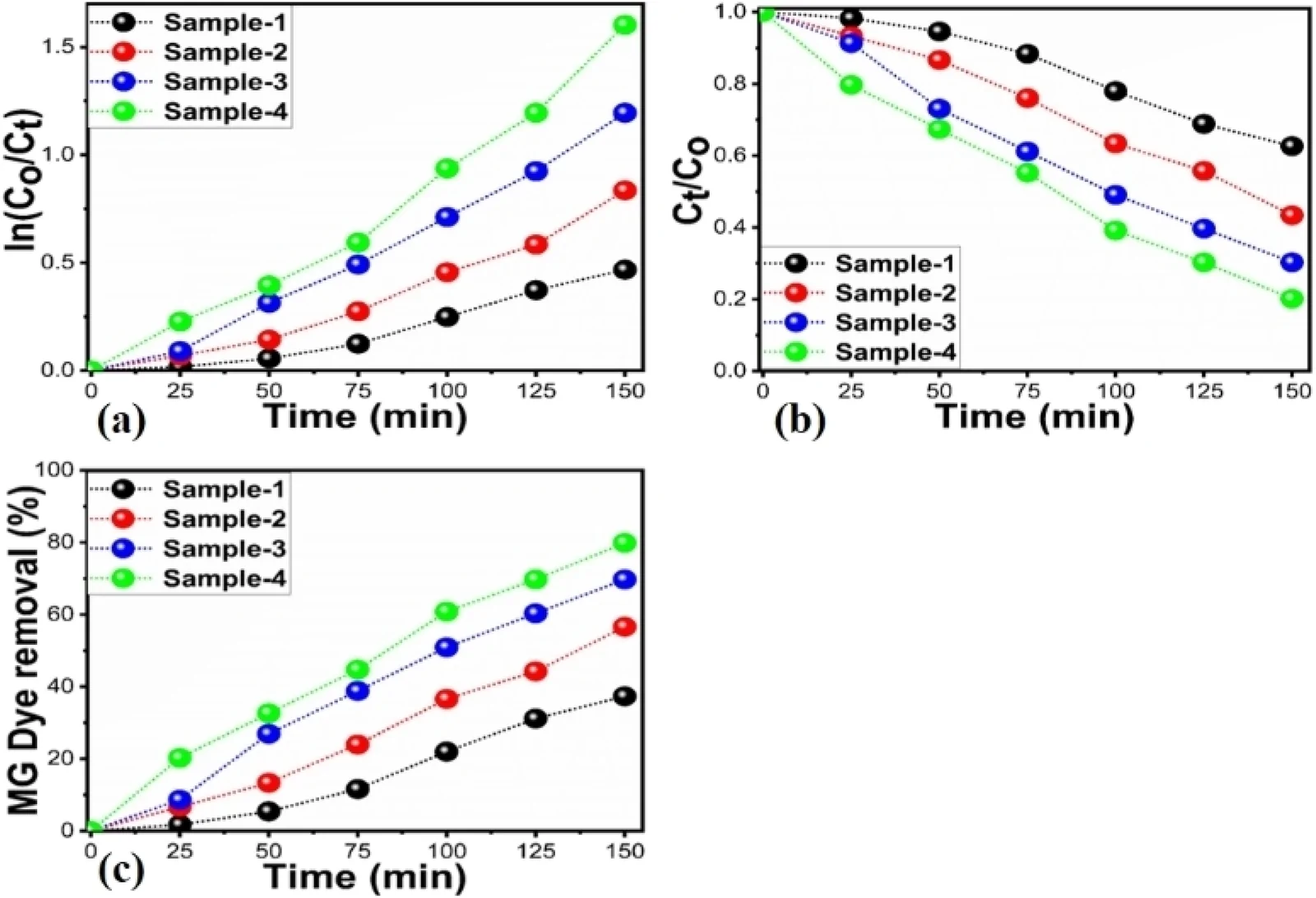
Degradation kinetics of datura mediated ZnO (sample 1 to sample 4) under the conditions of 10 mg catalyst dose and dye 8.2 × 10^−5^ M under the irradiation of natural of sunlight (a) pseudo first kinetic plot, (b) degradation rate, (c) dye removal %.

### 3.3. Photocatalytic performance of datura leaves extract mediated ZnO nanoparticles under the influence of initial dye concentration and catalyst dose

The effect of catalyst dose of 5 mg, 10 mg and 15 mg of sample 4 of datura leaves extract modified ZnO in 3.8 × 10^−5^ M dye solution was studied. Both the catalyst dose and dye concentration are affecting the dye degradation rate and removal %, hence they are called as the performance evaluation parameters for the new proposed photocatalytic materials. The natural sunlight exposure was given for 60 min and absorption spectra of MG were measured during 60 min as shown in Fig. 9a–c. A dye concentration of 3.8 × 10^−5^ M in the presence of 10 mg of catalyst dose of sample 4 of modified ZnO has shown relatively abrupt decrease in the absorbance value compared to the absorbance value at high concertation as shown in Fig. 9d. This clearly demonstrated the effect of initial dye concentration of the photocatalytic activity of modified ZnO towards the degradation of MG. Thus, the dye degradation rate is highly dependent on the dye concentration. Furthermore, a catalyst dose of 5 mg, 10 mg and 15 mg has revealed the robotic decrease in the absorbance value of MG as shown in Fig. 9c. This is attributed to the few dye molecules on the abundant surface sites of sample 4 of ZnO, thereby efficiently dye degradation took place. A low catalyst dose of 5 mg and 10 mg could have limited surface catalytic sites, hence the decrease in the absorbance value was limited, therefore the dye degradation rate could not be significant as shown in Fig. 9a and b. The initial dye concentration and catalyst dose studies have verified that both parameters are very crucial for the effectiveness evaluation of proposed photocatalytic material based on datura mediated ZnO (sample 4). The pseudo first order equation was again followed by the degradation of MG under the influence of different catalyst dose and low dye concentration as shown in Fig. 9d. It was found that the rate constant measured for the degradation of MG under the influence of 15 mg was higher 5.15 × 10^−2^ min^−1^ than the catalyst dose of 5 mg and 10 mg as given in Table 1. This suggested that the dye decomposition rate was highly accelerated and almost complete degradation was taking place with the use of 15 mg of catalyst dose of sample 4 of datura modified ZnO. The degradation rate of MG was predominated using 15 mg catalyst dose compared to 10 mg and 5 mg catalyst dose of sample 4 as shown in Fig. 9e. It could be seen that dye was swiftly degraded. The dye removal % was also examined under the effect of initial dye concentration and catalyst dose as shown in Fig. 9f. It was observed that the catalyst dose of 15 mg resulted dye removal 96.9%. The effect of initial dye concentration and catalyst dose confirmed that the dye degradation is highly influenced by the variability in the dye concentration and catalyst dose and such observations have been previously reported.^54^ Moreover, the dye removal of % of MG *via* adsorption was examined under magnetic stirring in dark conditions using 15 mg catalyst dose of pure ZnO and sample 1 to sample 4 in 3.8 × 10^−5^ M as shown in Fig. S1. The resultant analysis indicated the dye removal % as 5.7%, 9.3%, 11.6%, 14.7%, and 17.8% of MG *via* adsorption on pure ZnO, sample 1, sample 2, sample 3, and sample 4 respectively. These results suggested that the major dye removal % could be found under the photocatalytic degradation, and the datura leaves extract has potentially enhanced the photocatalytic performance of ZnO towards the degradation of MG.

**Fig. 9:**
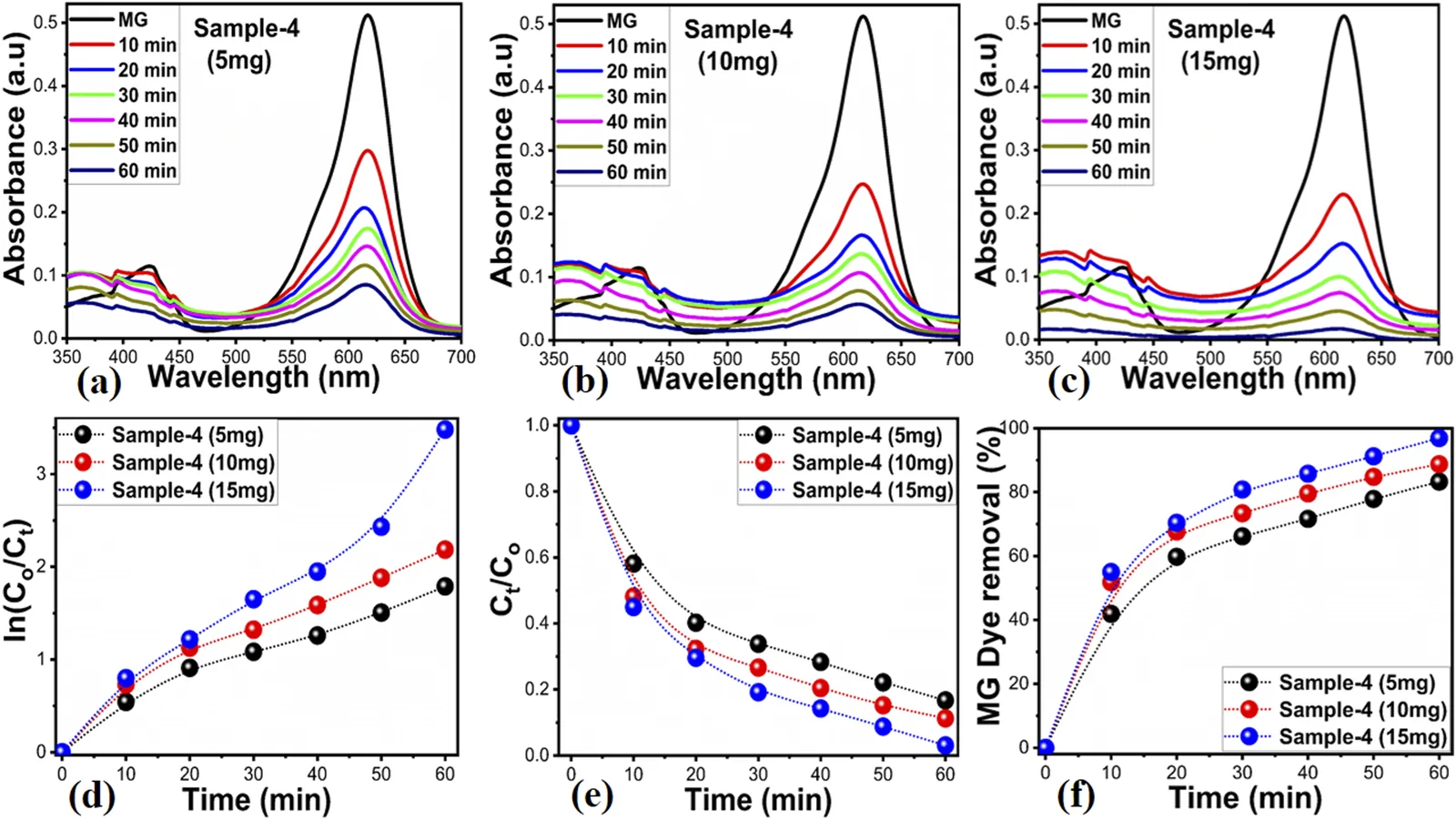
(a–c) UV-visible absorption spectra of MG in the presence of different catalyst dose of 5 mg, 10 mg and 15 mg catalyst dose datura mediated ZnO (sample 4) in dye 3.8 × 10^−5^ M under the irradiation of natural of sunlight, (d) pseudo first kinetic plot, (e) degradation rate, (f) dye removal %.

The efficiency of dye degradation is significantly affected by the pH of the dye solution,^55,56^ hence the effect of pH of MG dye solution was investigated as shown in Fig. 10a–d. Different pH values such as 3, 6, 9 and 12 were adjusted in 3.8 × 10^−5^ M of MG using 2 M HCl and 2 M NaOH and each solution contained 15 mg of catalyst dose of sample 4 of datura mediated ZnO. Then, these pH-adjusted dye solutions carrying 15 mg of catalyst dose were exposed to natural sunlight, thereby UV-visible absorption spectra recorded at different intervals of time as shown in Fig. 10a–d. The observations based on the different pH values through the analysis of absorption spectra clearly described the impact of pH of dye solution and the dye degradation was highly favored with increasing pH from 3 to 12 as shown in Fig. 10a–d. The catalyst dose and dye concentration are constant, the degradation of MG with increasing pH was mainly attributed to the effect of pH of reaction media and such results have been reported in the existing literature.^57–60^ The effect of pH on the degradation depends on the cationic and anionic nature of dye. It has bene observed that pH of zero-point charge (pHZPC) is one of the key elements in different fields such as adsorption, catalyst, wastewater and material engineering because it supports exploring the surface features of materials and determining their activity in different applications. Generally, it is believed that the pHZPC of ZnO is observed between 8.7 and 9.3 that depends on the size and synthetic method used for the proposed material. Below the pHZPC, the ZnO surface could be positively charged and MG being the cationic in nature has faced electrostatic repulsion with the surface of ZnO, hence the degradation of MG was found to be limited in acidic pH value of 3 and 6 as shown in Fig. 10a and b. At acidic pH, the existence of more protons and photogenerated hydroxyl radicals could combine neutral water molecules which may slow down the degradation of MG in acidic pH 3 and 6 owing less abundance of hydroxyl radicals.^61,62^ However, the surface of ZnO above to pHZPC could be negative, hence it can experience the electrostatic attraction between the cationic nature of MG, therefore the dye degradation was efficiently demonstrated in the alkaline pH 12 as shown in Fig. 10d compared to pH 3, and 6, the degradation rate of MG as higher in pH 9 as shown in Fig. 10c.

**Fig. 10:**
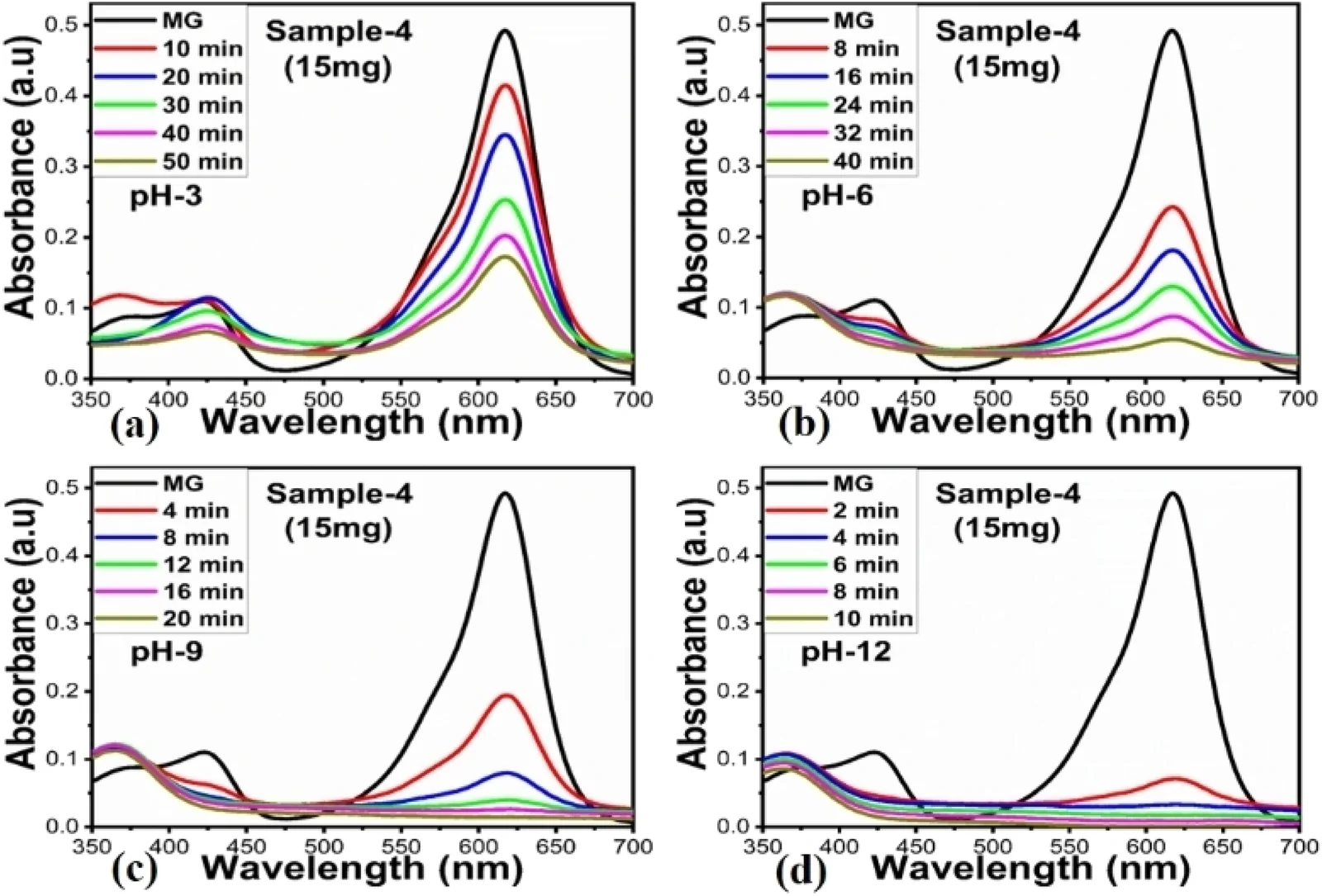
(a–d) UV-visible absorption spectra of MG in the presence of catalyst dose of 15 mg of datura mediated ZnO (sample 4) in dye 3.8 × 10^−5^ M having adjusted pH of 3, 6, 9, 12 under the irradiation of natural of sunlight.

Moreover, in the alkaline media the higher value of hydroxyl radicals could also support the degradation of MG at pH 12. The pH-based degradation of MG through the absorption spectra indicated the effect of pH on dye degradation and it was highly favored in the alkaline pH 12. The effect of pH of dye solution was further investigated for understanding the dye degradation kinetics as shown in Fig. 11a and b. It was observed that the dye degradation was following the pseudo first order equation and resultant rate constant were highly dependent on the pH of reaction medium as shown in Table 1. The pH 12 of dye solution has given out the largest value of rate constant 5.82 × 10^−1^ min^−1^. However, pH 9, 6 and 3 dye solution has shown successive decrease in the rate constant value as given in Table 1. The degrading rate of MG was highly affected by the pH of reaction media as shown in Fig. 11b, and it was seen that the dye was rapidly degraded with a short interval of 10 min that has again indicated the degradation of MG in the presence of ZnO was highly strengthened by the alkaline pH 12. The dye removal 99.8% was found for the pH 12 which is higher than pH 9, 6 and 3 as shown in Fig. 11c. The dye removal % analysis has revealed that the negatively surface ZnO based catalyst favored the MG degradation at pH 12 due to the efficient electrostatic attraction between the negative surface of ZnO and positively charge MG which could cause the frequent interaction, consequently accelerated the decomposition of dye.

**Fig. 11:**
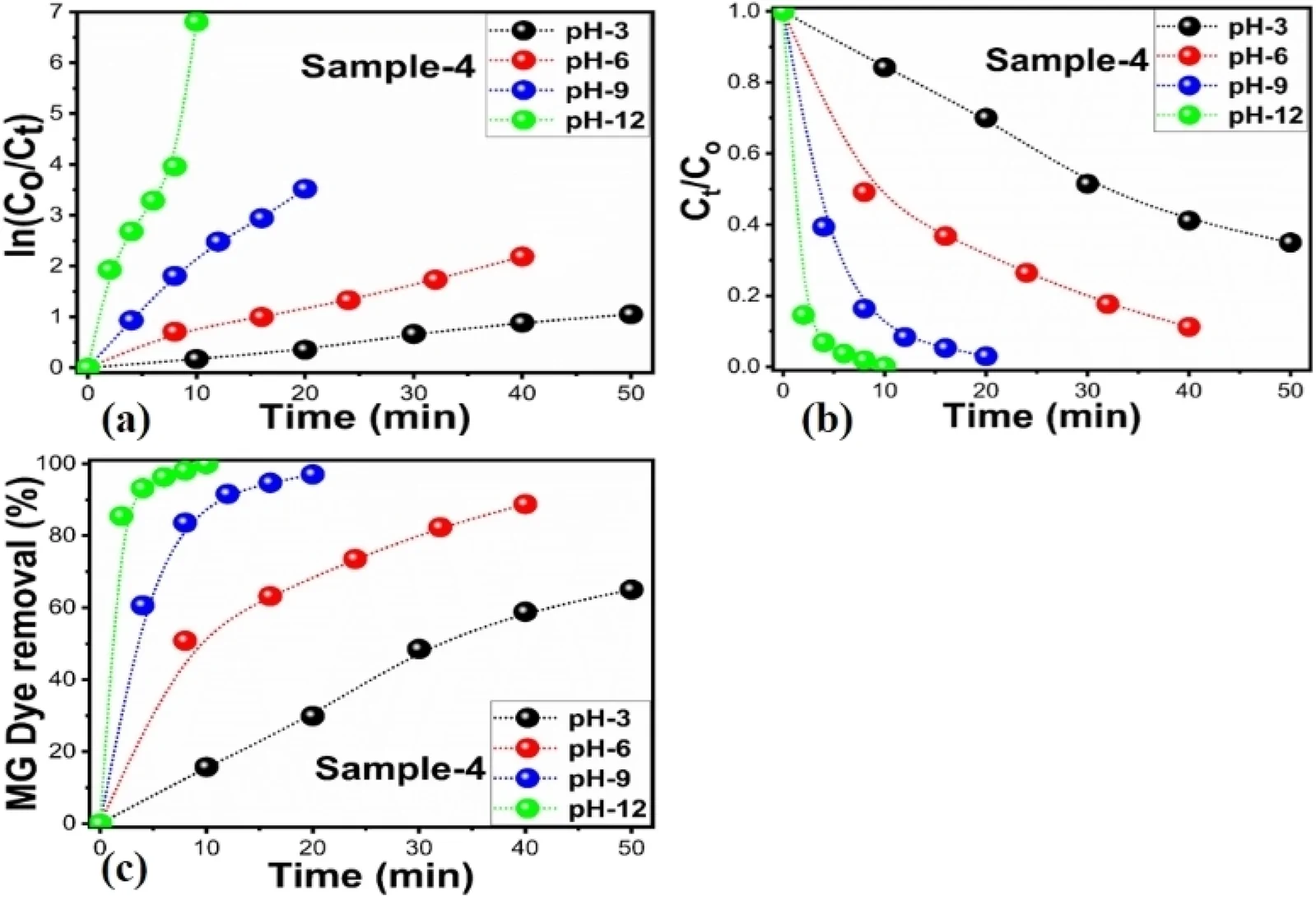
Degradation kinetics under the influence of different pH of reaction media (a) pseudo first kinetic plot, (b) degradation rate, (c) dye removal %.

Likewise, the photocatalytic activity of as produced ZnO materials with datura leaves extract was studied towards MB under the illumination of natural sunlight in aqueous solution. Using 15 mg catalyst dose of pure ZnO, sample 1, sample 2, sample 3 and sample 4 of datura mediated ZnO in MB concentration of 2.81 × 10^−5^ M was exposed for 75 min under the illumination of natural sunlight. Before the photocatalytic measurements, the dye removal % of MB was studied under the magnetic stirring in dark for the estimation of MB on the prepared materials as shown in Fig. S1. It was seen that relative adsorption of MB in the dark using only magnetic stirring was observed for pure ZnO, sample 1, sample 2, sample 3 and sample 4 as 4.8%, 6.7%, 9.2%, 12% and 14.2% respectively. This analysis has illustrated that under the magnetic stirring in dark the dye removal % was limited. The UV-visible absorbance spectrum was measured with different intervals of time as shown in Fig. S2. It could be seen that the pure ZnO possessed as poor photocatalytic towards photodegradation of MB because with the passage of degradation time, there was negligible decrease in the absorbance value. However, for the datura leaves extract mediated ZnO resulted in significant decline in the absorbance value as described in Fig. S2. The pseudo first order kinetics equation was employed to examine the degradation kinetics of MB under the influence of 15 mg of catalyst dose of pure ZnO, sample 1, sample 2, sample 3 and sample 4 of datura mediated ZnO and corresponding rate constant value is shown in Table 2. It is seen than pure ZnO showed a low-rate constant value of 6.28 × 10^−3^ min^−1^ compared to the rate constant value of 2.64 × 10^−2^ min^−1^ for sample 4 of ZnO synthesized with datura leaves extract, confirming that the material has high potential to degrade the synthetic dyes effectively in aqueous. The dye removal % was also evaluated and the sample 4 ZnO resulted 88.4% MB removal percentage as given in Table 2. Also, the effect of pH of dye MB solution was studied using 2.81 × 10^−5^ M dye concentration adjusted with 2 M HCl and NaOH having aa pH value of 4, 8 and 12, and using 15 mg catalyst dose of sample 4 of ZnO prepared with datura leaves extract, then UV-visible absorbance spectra were taken as shown in Fig. S3. The observations about the decrease in the absorbance value of MB under the different pH of dye solution indicated that the absorbance was decreasing speedily in alkaline pH 12 than pH 8 and pH 4, again verifying that the surface of ZnO was negatively charged in alkaline pH and MB being cationic dye was largely adsorbed on the surface of ZnO, thereby rapid dye degradation was observed as shown in Fig. S3. The degradation kinetics under the influence of pH of dye solution was studied and pseudo first order kinetics was filled by the degradation of MB and estimated rate constant values of MB using sample of ZnO are given in Table 2. It could be observed that the rate constant was higher in pH 12 than pH 4, indicating the dye degradation rate was highly favorable in pH 12. The dye removal % was calculated under the influence of pH of dye solutions such as 4, 8 and 12 as given in Table 2. It was seen that the dye removal % was about 98.3% in pH 12 which is relatively higher than pH 8 and 4. The inefficiency of dye removal in acidic pH 4 could be attributed to positive surface of ZnO which further created strong repulsion for the cationic MB dye, thereby lesser interaction may be occurring, consequently dye removal was limited in pH 4. Furthermore, the dye removal % was investigated using 15 mg catalyst dose datura leaves extract mediated ZnO (4) at neutral pH 7 for MG dye 3.8 × 10^−5^ M and MB in 2.81 × 10^−5^ M under the illumination of sunlight as shown in Fig. S4. The removal % for MG was noticed around 92.7% in 3.8 × 10^−5^ M, whereas removal % for MB was seen in 62.2% in 2.81 × 10^−5^ M. Likewise, MG dye 3.8 × 10^−5^ M and MB in 2.81 × 10^−5^ M were adjusted with pH 12 and containing 15 mg catalyst dose of sample 4, and their dye removal % was analyzed in the dark. The limiting dye fading % for MG and MB were found 51.6% and 32.2% respectively.

Performance observations about pure ZnO and modified ZnO samples towards MB photodegradation

**Table:** 

Samples (ID)	Amount of dye (MB)	Time (min)	Degradation (%)	Rate constant (*K*)
Pure ZnO (15 mg)	2.81 × 10^−5^ M	75	36.8	6.28 × 10^−3^
Sample-1 (15 mg)			51.7	9.28 × 10^−3^
Sample-2 (15 mg)			59.2	1.21 × 10^−2^
Sample-3 (15 mg)			65.1	1.39 × 10^−2^
Sample-4 (15 mg)			88.4	2.64 × 10^−2^

**Table:** 

Sample (ID)	pH (value)	Time (min)	Degradation (%)	Rate constant (*K*)
Sample-4 (15 mg)	pH-4	75	19.1	2.69 × 10^−3^
	pH-8	35	87.2	5.68 × 10^−2^
	pH-12	15	98.3	2.81 × 10^−1^

### 3.4. Radical trapping and cycling stability measurements

To understand the nature of reactive species involved in the photocatalytic degradation of MG, a scavenger measurement was carried out. Different trapping agents such as disodium ethylenediaminetetracetate (EDTA-2Na), silver nitrate and isopropyl alcohol (IPA) used a concentration of 20 ppm of each scavenger agent in the 3.8 × 10^−5^ M dye solution with a catalyst dose of 15 mg of sample 4 of datura mediated ZnO. The dye removal % in the presence of trapping agents is shown through bar graph representation as shown in Fig. 12a. The trapping agent EDTA-2Na is responsible for trapping photogenerated holes, silver nitrate is responsible for trapping photogenerated electrons and IPA can trap different reactive species such as OH˙^−^, and O_2_˙^−^. It was observed that the EDTA-2Na was largely decreased the dye removal %, confirming the photogenerated holes were the main reactive species involved in the photodegradation of MG followed by photogenerated electrons and different oxide radicals such as OH˙^−^, and O_2_˙^−^. The scavenger measurement has indicated that these trapping agents block the degradation reaction through quenching highly reactive intermediates which are found to be responsible for the degradation of MG. The relative decrease in the dye removal % was noticed in the order as EDTA-2Na > IPA > AgNO_3_ as shown in Fig. 12a.

**Fig. 12:**
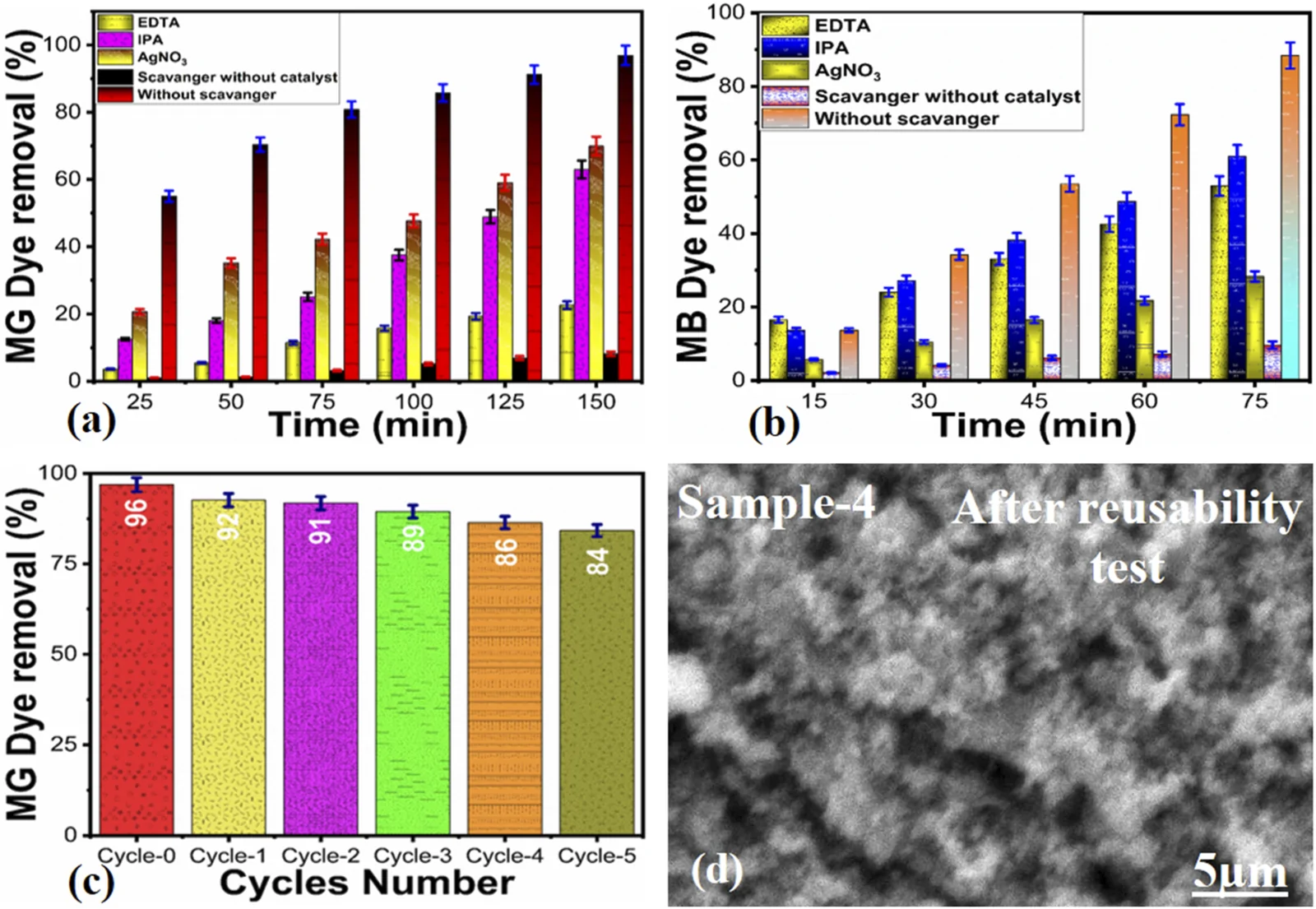
(a) MG removal % under the influence of different scavenger agents, (b) MB removal % in the presence of various scavenging substances (c) cycling stability of sample 4 of datura mediated ZnO using different MG photodegradation measurements, (d) SEM image after the cycling measurements.

Similarly, the above scavengers were added MB concnertaion of 2.81 × 10^−5^ M, catalyst dose of 15 mg of sample of ZnO and 20 ppm of each scavenger in separate experiment. The corresponding dye removal % was estimated after degradation analysis as shown in Fig. 12b. Herein, the MB was strongly inhibited using silver nitrate which is electron scavenger agent. In dye solution when dissolved oxygen interacts with photogenertaed electrons thereby super oxide are produced which are participating in the MB degradation and such observations have been reported in previous study.^63^ Hence, scavenger analysis suggested that the degradation of MB was suppressed by silver nitrated followed by EDTA and NaBH_4_, and the main radical is the superoxide for the degradation of MB.

To verify the reuse applicability of sample 4 of dutra mediated ZnO during the photodegradation of MG, recycling measurements were performed 3.8 × 10^−5^ M dye solution with a catalyst dose of 15 mg. After each measurement, the particles were collected by centrifugation at 5000 rpm and washed several times with deionized water followed by ethanol. After drying, these collected particles were again used for the photodegradation of MG and same process was repeated 5 times. The corresponding dye removal % during each cycling test is shown in Fig. 12c. The dye removal % during the 5 cycles was slightly decreased that could be assigned to negligible loss of ZnO nanoparticles and maybe some of the dye molecules could be adsorbed to surface of ZnO. The cycling performance of datura mediated ZnO has revealed that the photocatalytic material has shown improved stability and its can be used several times, keeping in view the careful collection and washing of reused ZnO nanoparticles. Moreover, the SEM analysis was performed on the reused particles as shown in Fig. 12d, confirming the morphology of ZnO remained unchanged after 5 cycling measurements for the photodegradation of MG. To support the SEM results in terms of structural changes for sample 4 after the cycling test, FTIR analysis was performed and obtained spectrum is shown in Fig. S5. It could be seen that the major IR bands at 472 and 564 cm^−1^ were observed and they were assigned to Zn–O stretching vibrations, indicating that ZnO retained its chemical structure and consequently it has shown structural integrity even after cycling test. In addition to this, the photocatalytic performance of datura leaves extract mediated ZnO (sample 4) was performed in the tape water using 15 mg catalyst dose in 2.81 × 10^−5^ M of MB as shown in Fig. S6. The absorbance curves were measured for 90 min and kinetics of dye degradation were also studied as shown in Fig. S6a–c. The dye removal % was observed in the tape water for the MB and it was estimated to be around 82.7%. The photocatalytic performance of sample 4 of ZnO for MB in tape water verified that it can be used as a promising and alternative photocatalytic material for the real-world wastewater treatment.

### 3.5. Electrochemical performance of datura leaves extract mediated ZnO towards dye detection

Besides the photocatalytic performance of as produced ZnO using datura leaves extract, they were examined in terms of electrochemical activity for the detection of MG and MB in 0.1 M NaOH electrolytic solution. For performing preliminary electrochemical study, CV curves for bare glassy carbon electrode (BGCE) pure ZnO, sample 1, sample 2, sample 3 and sample 4 of datura leaves extract mediated ZnO were recorded at a scan rate of 50 mV s^−1^ in 0.1 mM MB solution as shown in Fig. 13a. It was seen that pure ZnO showed negligible electrochemical activity, however the datura leaves extract mediated ZnO (sample, sample 2, sample 3 and sample 4) has shown obvious redox peaks as shown in Fig. 13a. It was observed that sample 4 of ZnO was found to be highly electrochemically active for the detection of MB because the oxidation peak is well resolved and carried the significant amount of peak current. The oxidation potential of MB was found to be −0.24 V and it was seen in good agreement with the reported work.^64^ The phytochemicals from datura leaves extract not only enhanced the photocatalytic properties of ZnO, but they have potentially improved the electrochemical activity of ZnO *via* surface modification, size alteration and energy band gap variability. The CV curve of sample 4 of ZnO was measured in 0.1 mM MB at 50 mV s^−1^ compared with CV curve of sample 4 only in 0.1 M NaOH electrolytic solution as shown in Fig. 13b. It was clearly viewed that the sample 4 has mainly showed sensitive signal for MB and the oxidation peak was only contributed form the oxidation of MB as shown in Fig. 13b. Likewise, sample 4 of ZnO was utilized in the 0.1 mM MG and corresponding CV cure was measured as shown in Fig. 13b. It was seen that sample 4 of ZnO has shown negligible electrochemical redox behaviors. The electrochemical performance of nanomaterials is usually selective in the response towards the oxidation or reduction of the substance. In terms of redox potential, MB requires low oxidation potential to be oxidized, while MG needs large oxidation potential, hence the oxidation of MB was favored in the presence of datura mediated ZnO. Despite MB is relatively more chemically stable than MG. The surface of datura mediated ZnO could be modified in a way that selectively oxidizes the MB; hence it was inactive for the oxidation of MG.

**Fig. 13:**
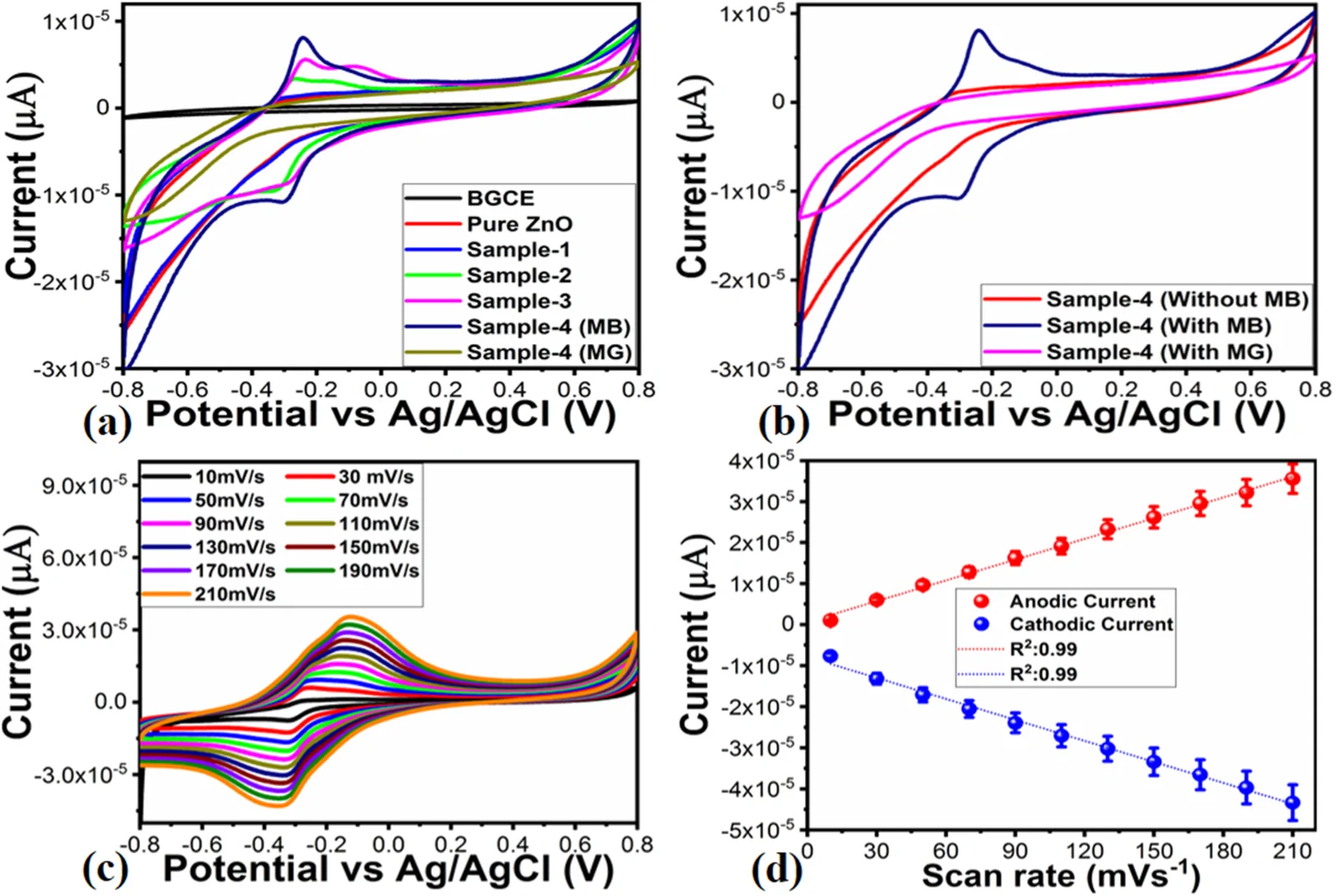
(a) CV analysis of different modified electrode materials at a scan rate of 50 mV s^−1^ in 0.1 mM MB in 0.1 M NaoH, (b) CV performance of sample 4 of ZnO in the presence of 0.1 mM MB and MG and without MB concentration, (c) CV cycles at different scan rates in 0.1 mM MB, (d) linear plot of anodic peak and cathodic peak currents against different scan rate.

Based on the preliminary CV analysis for both MB and MG, samples 4 of ZnO was further applied to describe the modified electrode kinetics, detection of different MB concentrations, electrochemical active surface area and charge transfer resistance. The influence of scan rate on the peak potential and peak current of MB in 0.1 mM was examined using CV analysis. CV curves at different scan rates were recorded as presented in Fig. 13c. It was observed that there was successive increase in the redox peak current with increasing scan rate and at the same time, there was shift for anodic peak towards positive potential and cathodic peak towards negative potential as shown in Fig. 13c. The peak potential shifts could be attributed to improved electrochemical performance of sample 4 of ZnO which is controlled by diffusion process.^65,66^

Furthermore, the diffusion-controlled process was strengthened by the plot of peak current against square root of scan rate as shown in Fig. 13d. It was seen that the peak currents were successively increased linearly against square root of scan rate as described through regression coefficient of (*R*^2^ = 0.99), revealing the electrochemical reaction on the surface of modified electrode is diffusion controlled.

Keeping in view the slow nature of CV technique, conventional chronoamperometry was employed at fixed potential of 0.25 V *versus* Ag/AgCl for the estimation of linear range of MB detection. The measured chronoamperometry response time curves in different MB concentrations are shown in Fig. 14a. Chronoamperometry response time analysis has shown the rise in the current with addition of specific MB concentration, and the successive additions were done from 0.005 mM to 4 mM as shown in Fig. 14a. The steady response time in 0.01 mM MB was observed to be approximately 30 s. The linear plot was formulated by picking oxidation peak current of each MB against different concentrations as shown in Fig. 14b. The linear plot has shown excellent analytical performance for the MB concentration detection from 0.005 mM to 4 mM as supported by regression coefficient of (*R*^2^ = 0.99). The sensitivity of 8.847 µA mM^−1^ cm^−2^ was observed for the presented amperometric method.

**Fig. 14:**
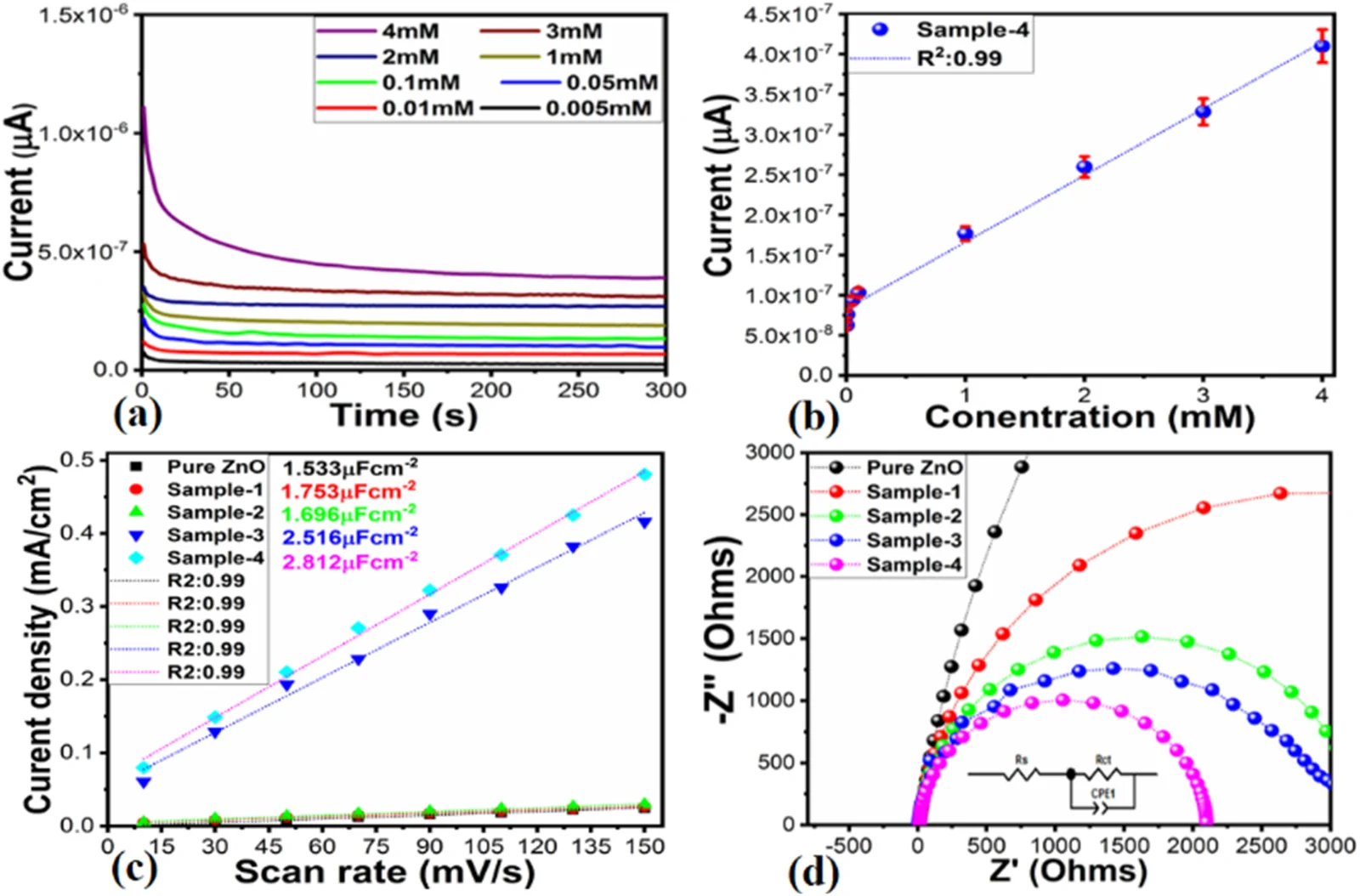
(a) Chronoamperometry response time cures in various MB concentrations using fixed potential of 0.25 V *vs.* Ag/AgCl, (b) linear plot of prdocued current against MB concentrations, (c) ECSA estimation of different materials including pure ZnO, sample 1, sample 2, sample 3 and sample 4 of datura leaves extract mediated ZnO *via* linear plot current density from non-faradic CV curves *vs.* different scan rate, (d) Nyquist plots.

These wide range for the detection of MB by sample 4 of ZnO could contribute to enhanced electrochemical activity through surface modification, particle size and optical band gap engineering using datura leaves extract. The limit of detection (LOD) was further estimated through exiting literature.^67^ The LOD performance was seen with a value of 0.0005 mM, revealing the capability of modified electrode for the detection of MB. The sample 4 of ZnO has shown enhanced electrochemical activity in terms of wide linear range and low LOD due to abundant and accessible surface-active sites, rapid charge transfer kinetics, and high electroactive surface area. To support the enhanced electrochemical performance of sample 4 of ZnO for MB detection, the electrochemical surface area was examined using non-faradic region in the CV measurements at various scan rates as shown in Fig. S7. Typically, the potential window was used between −0.2 V to 0.2 V *versus* Ag/AgCl was applied to pure ZnO, sample 1, sample 2, sample 3 and sample 4 of datura mediated ZnO. It coild be seen that the CV curves recorded at various scans possessed good non-faradic behavior. Then, the *C*_dl_ was calculated using the difference of anodic and cathodic current density followed by plotting the obtained against scan rate as show in Fig. 14c. The calculation of *C*_dl_ was perfumed as per reported work.^68^ The linear plot has shown corresponding slope values, 1.533 µF cm^−2^, 1.753 µF cm^−2^, 1.696 µF cm^−2^, 2.516 µF cm^−2^ and 2.612 µF cm^−2^ were assigned to the *C*_dl_ of each material. The sample 4 of ZnO has shown larger *C*_dl_ value compared to sample 3, sample 2, sample 1 and pure ZnO, indicating the high electrochemical active surface experienced by the sample 4, hence it has shown improved electrochemical activity. The charge transfer resistance characterization was performed for the validation of high performance of sample 4 of ZnO towards the MB detection. For this purpose, pure ZnO, sample 1, sample 2, sample 3 and sample 4 of datura leaves extract mediated ZnO were examined using EIS measurements in the sweeping frequency range of 100 kHz to 0.1 Hz, an amplitude of 5 mV and oxidation potential of MB as biasing potential. The experimental EIS data was further treated with z-view software for simulation and finding the well fitted equivalent circuit as shown in inset of Fig. 14d. The Nyquist plots for the different modified electrodes shown in Fig. 14d representing the obvious information about the charge transfer resistance as could be viewed from the arc of each plot.^69^ From this rapid view, it was seen that sample 4 of ZnO was shown the lowest arc area, thus it has the lowest charge transfer resistance. Generally, it is believed that the smaller semicircle of Nyquist plot, lower is charge transfer resistance, higher is electrical conductance. But the charge transfer resistance was calculated using fitted equivalent circuit as inset of Fig. 14d. The circuit was carrying three main elements such as capacitance double layer (*C*_dl_), solution resistance (*R*_s_) and charge transfer resistance (*R*_ct_). The equivalent circuit-based charge transfer analysis has given out the 23 133 ohm, 5900-ohm, 3199-ohm, 2630 ohm, and 2088 ohm for pure ZnO, sample 1, sample 2, sample 3, and sample 4 of ZnO respectively. The sample of 4 of ZnO has expressed the lowest *R*_ct_, therefore it experienced enhanced electrochemical activity.^70^ Both EIS and electrochemical active surface area studies have confirmed that the increased electrochemical performance of sample 4 of ZnO towards MB detection were attributed to increased active surface area and fast charge transfer kinetics.

The photocatalytic degradation mechanism of MG/MB in the presence of datura mediated ZnO nanoparticles was generally proposed as shown in Scheme 3. The degradation mechanism of dyes onto ZnO predominantly involves the advanced oxidation process.^71,72^ The dye degradation in the presence of semiconducting ZnO was initiated by the photo excitation that caused the generation of electron and hole pairs in the conduction and valence band respectively. The photogenerated electrons in the conduction band interact with dissolved oxygen molecules lying close to the surface of catalyst and form superoxide radicals (O˙^2−^). The photogenerated holes can produce hydroxyl radicals (OH˙) through the interaction with surface adsorbed OH^−^ groups.^73–77^ Species like O^2−^ can serve as oxidizing agents and offer additional sources of hydroxyl radicals. During the photodegradation process, hydrogen peroxide (H_2_O_2_) could be generated as an intermediate product, consequently it can take electrons to produce OH* radicals. Hence, these reactive species react with dye molecules and decomposing them into harmless products including CO_2_ and H_2_O. Additionally, the direct oxidation of absorbed organic pollutants on the surface of photocatalytic material have been observed.^73^ The possible reactions involved during the use of ZnO through the oxidative role of holes and reductive role of electrons for the degradation of MG/MB dye could be summarized as under.^74^

3 ZnO + *hν* → ZnO (e^−^ + h^+^) (photoexcitation causing generation of electrons and holes)

4 O_2_ + e^−^ → O_2_*^−^ (photogenerated electrons forming superoxide radical anion)

5 h^+^ + H_2_O → H^+^ + OH^−^ (photogenerated holes neutralize OH^−^ group into OH)

6 H_2_O_2_ + *hν* → 2OH* (photocatalysis induces hydroxyl radicals)

7 OH* + dye → CO_2_ and H_2_O (formation of harmless products)

**Scheme 3:**
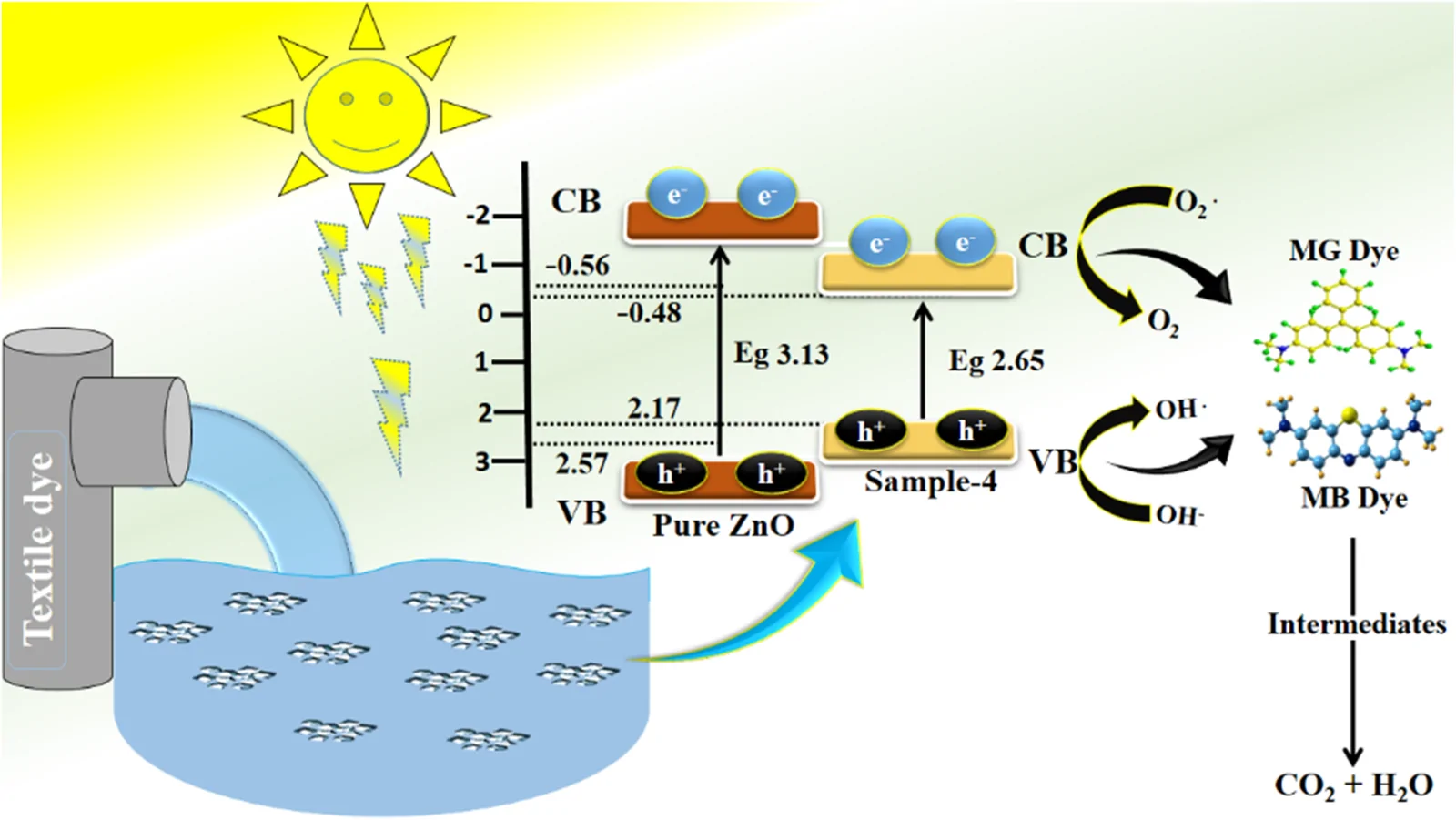
Proposed MG and MB reaction mechanism on the datura mediated ZnO.

The photocatalytic activity of datura mediated ZnO nanoparticles was compared with the recently designed photocatalytic materials for the degradation of MG as given in Table S2. Likewise, the comparative study of MB degradation with the recent literature is shown in Table S3. The superior dye removal %, facile, and low-cost nature of sample 4 of ZnO synthesized with datura leaves extract and it offers a promising strategy offer for scalable synthesize of photocatalytic materials towards the wastewater treatment applications.

## 4 Conclusions

In summary, this study presents a sustainable, phytochemical-assisted route for the synthesis of ZnO nanoparticles using datura leaf extract and demonstrates their effective application in the photocatalytic degradation of malachite green (MG) and methylene blue (MB) under natural sunlight irradiation. Systematic investigations reveal that photocatalytic performance is strongly governed by morphological features, defect structures, catalyst dosage, solution pH, and initial dye concentration. Notably, the sample 4 achieved a high degradation efficiency of 96.8% for a 3.8 × 10^−5^ M MG solution using 15 mg of catalyst. The degradation process was significantly enhanced under alkaline conditions (pH 12), highlighting the role of surface chemistry and reactive species generation. Furthermore, sample 4 exhibited excellent cycling stability, confirming its robustness and reusability for practical applications. The superior performance is attributed to the synergistic effects of phytochemical-mediated synthesis, which promotes controlled nanostructure formation, surface functionalization, and improved charge carrier dynamics. The sample 4 of ZnO was found highly electrochemically active towards the detection of MB and detection range was observed from 0.005 mm to 4 mM and low LOD of 0.0005 mM. The use of datura leaf extract as a natural source of reducing, capping, and stabilizing agent's offers a facile, low-cost, and environmentally benign strategy for the fabrication of high-performance ZnO-based nanomaterials. Overall, this work provides valuable insights into green synthesis approaches for advanced photocatalysts and highlights their strong potential in environmental remediation and solar-driven energy-related applications.

## Author contributions

Aslam Khan did material synthesis. Aneela Tahira did XRD analysis. Khalida Parveen Mahar, did supervision. Muhammad Ali Bhatti did photocatalytic tests. Elmuez Dawi edited the draft. Lama Saleem validated the results. Ryadh H. Alshamari visualized the results. Zafar Hussain Ibupoto wrote the first draft of manuscript and supervised the work. Ayman Nafady did the overall review of the data and manuscript before submission.

## Conflicts of interest

There was no conflict of interest in the presented research work.

## Supplementary Material

RA-OLF-D6RA07680G-s001

## Data Availability

The authors declare that the data supporting this study is available within the paper. Supplementary information (SI) is available. See DOI: https://doi.org/10.1039/d6ra07680g.
