# Supplementary material for: Improved electrochemical and photocatalytic activity of datura leaf extract mediate ZnO nanoparticles for the degradation of synthetic organic dyes
Source: RSC Adv. 2026 Oct 14;16(47):62735–53. doi: 10.1039/d6ra07680g (PMC13662650; doi:10.1039/d6ra07680g)
Supplement: Supplementary file 1 [file RA-016-D6RA07680G-s001.pdf]

Supplementary Information (SI) for RSC Advances.
This journal is © The Royal Society of Chemistry

Supplementary Information

Improved electrochemical and photocatalytic activity of Datura leaf extract mediate ZnO nanoparticles for the degradation of synthetic organic dyes

Aslam Khan[1], Aneela Tahira[1], Khalida Parveen Mahar[1], Muhammad Ali Bhatti[3], Elmuez Dawi[4], Lama Saleem[6], Riyadh H. Alshamari[5], Zafar Hussain Ibupoto[2*], Ayman Nafady[5*]

[1]Institute of Chemistry, Shah Abdul Latif University Khairpur Mirs, Sindh, Pakistan.

[2]Institute of Chemistry, University of Sindh, Jamshoro 76080, Pakistan.

[3]Centre for Environmental Sciences, University of Sindh Jamshoro, 76080, Sindh, Pakistan.

[4]College of Humanities and Sciences, Department of Mathematics and Sciences, Ajman University, P.O. Box 346, Ajman, UAE.

[5]Chemistry Department, College of Science, King Saud University, Riyadh, 11451, Saudi Arabia.

[6]Biomolecular Science, Earth and Life Science, Amsterdam University, 1081 HV Amsterdam, Netherlands.

[*]Corresponding author(s): Zafar Hussain Ibupoto, PhD, Email : zaffar.ibhupoto@usindh.edu.pk, orcid.*org/0000-0002-6756-9862*

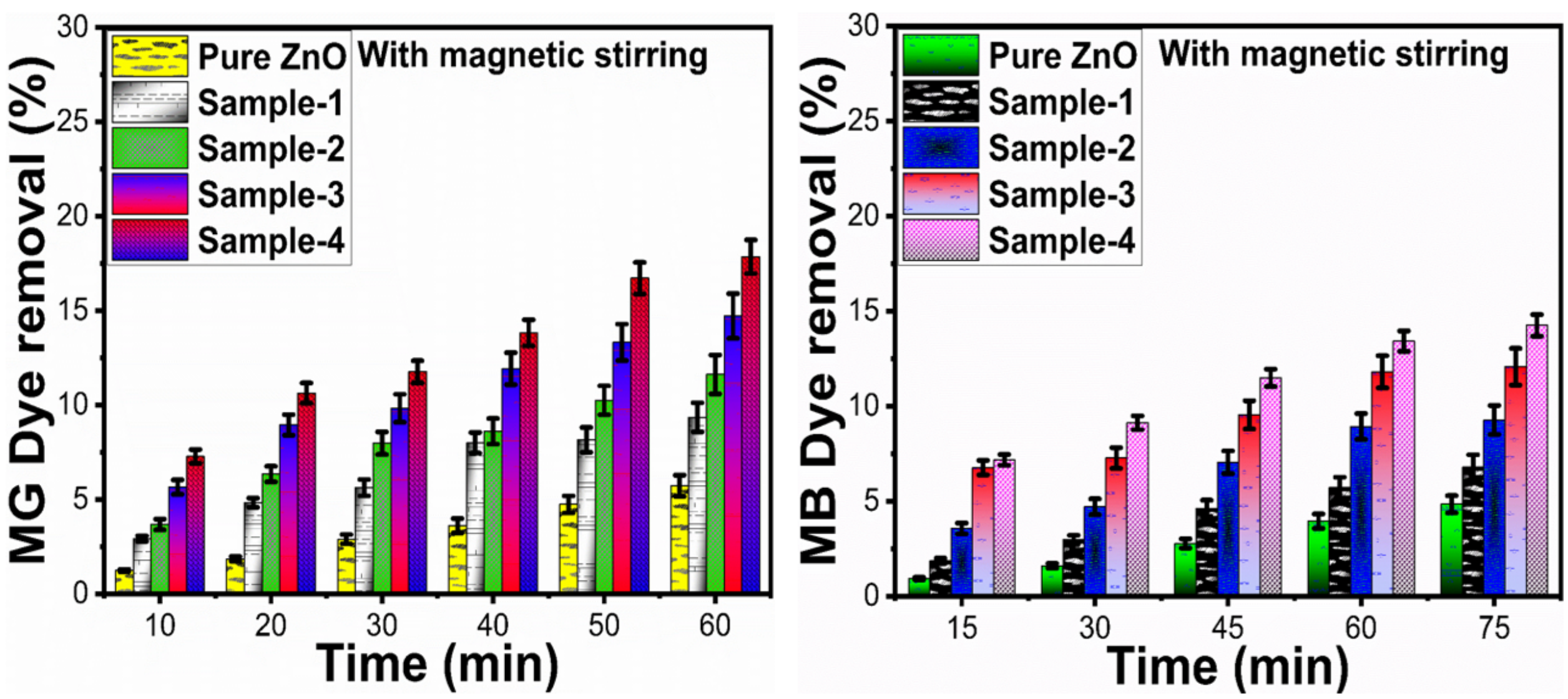

Figure (S1): Dye removal % of MG and MB under the magnetic stirring in dark

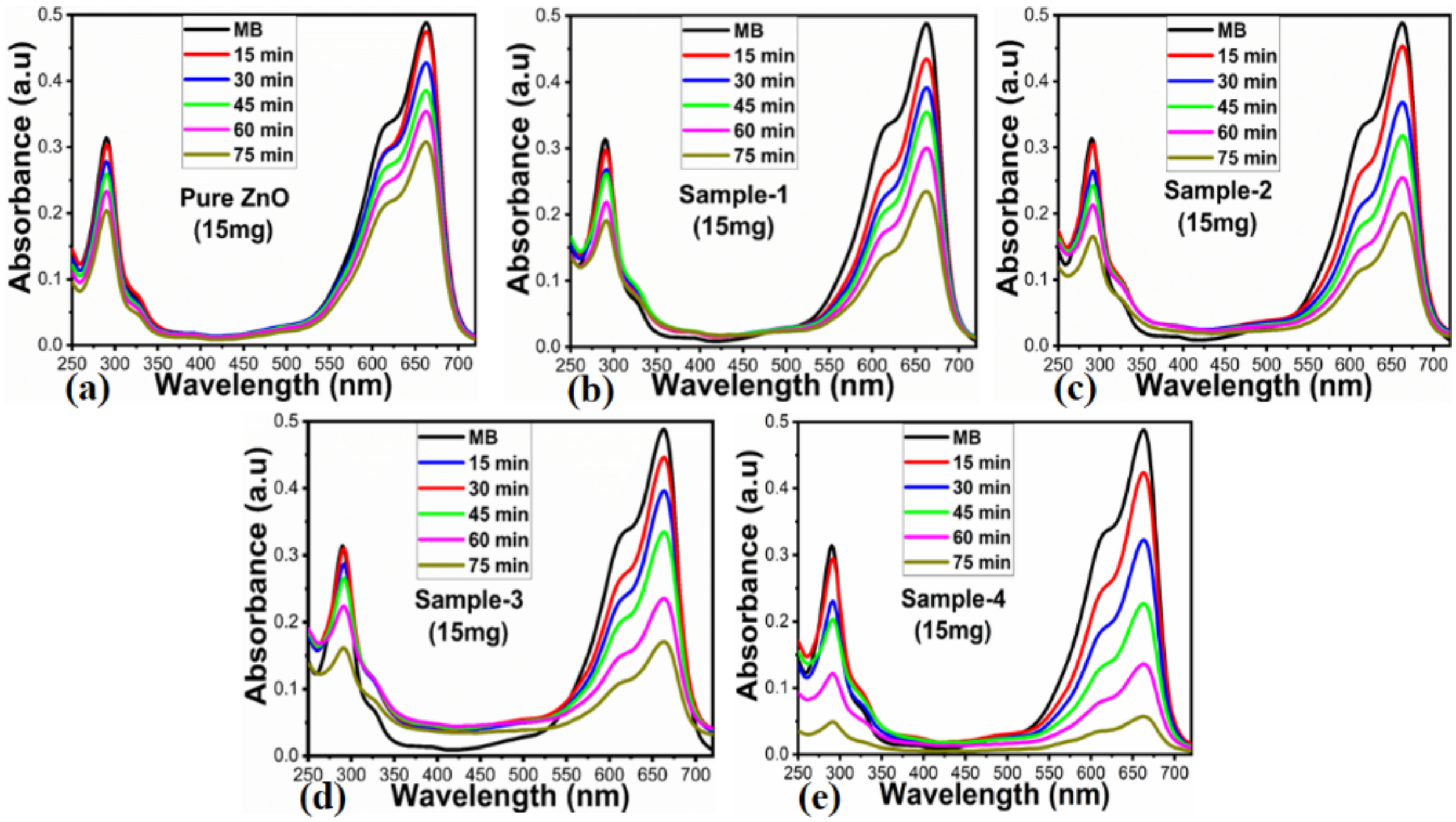

Figure (S2): UV-visible absorbance spectra of MB 2.81 × 10⁻⁵M using 15mg photocatalyst dose under the illumination of natural sunlight (a) pure ZnO, (b-e) Datura leaves extract mediated Zn

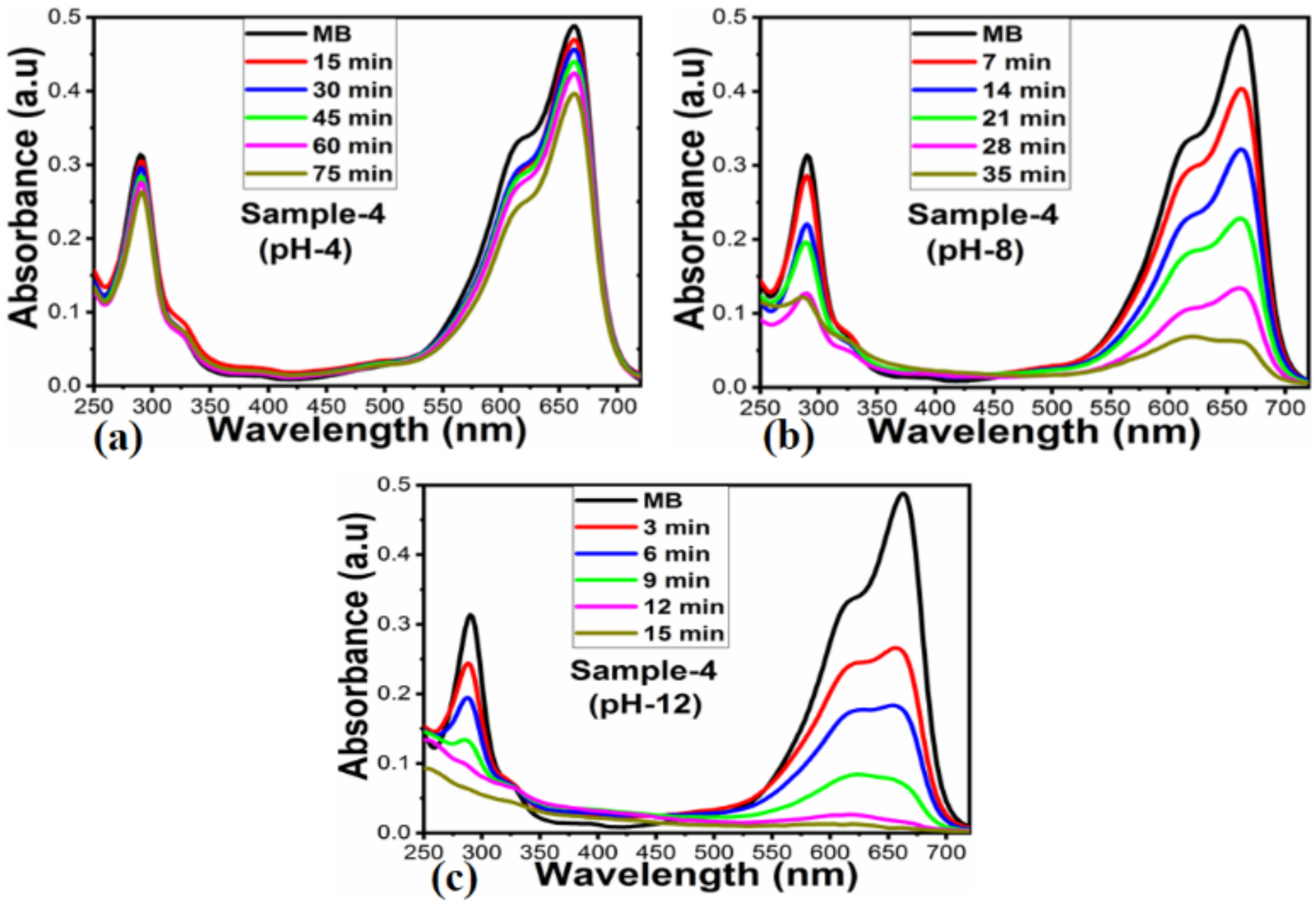

Figure (S3): UV-visible absorbance spectra of MB 2.81 × 10⁻⁵M using 15mg photocatalyst dose of sample 4 of Datura mediated ZnO under the illumination of natural sunlight with adjusted pH value (a) 4, (b) 8 and (c) 12

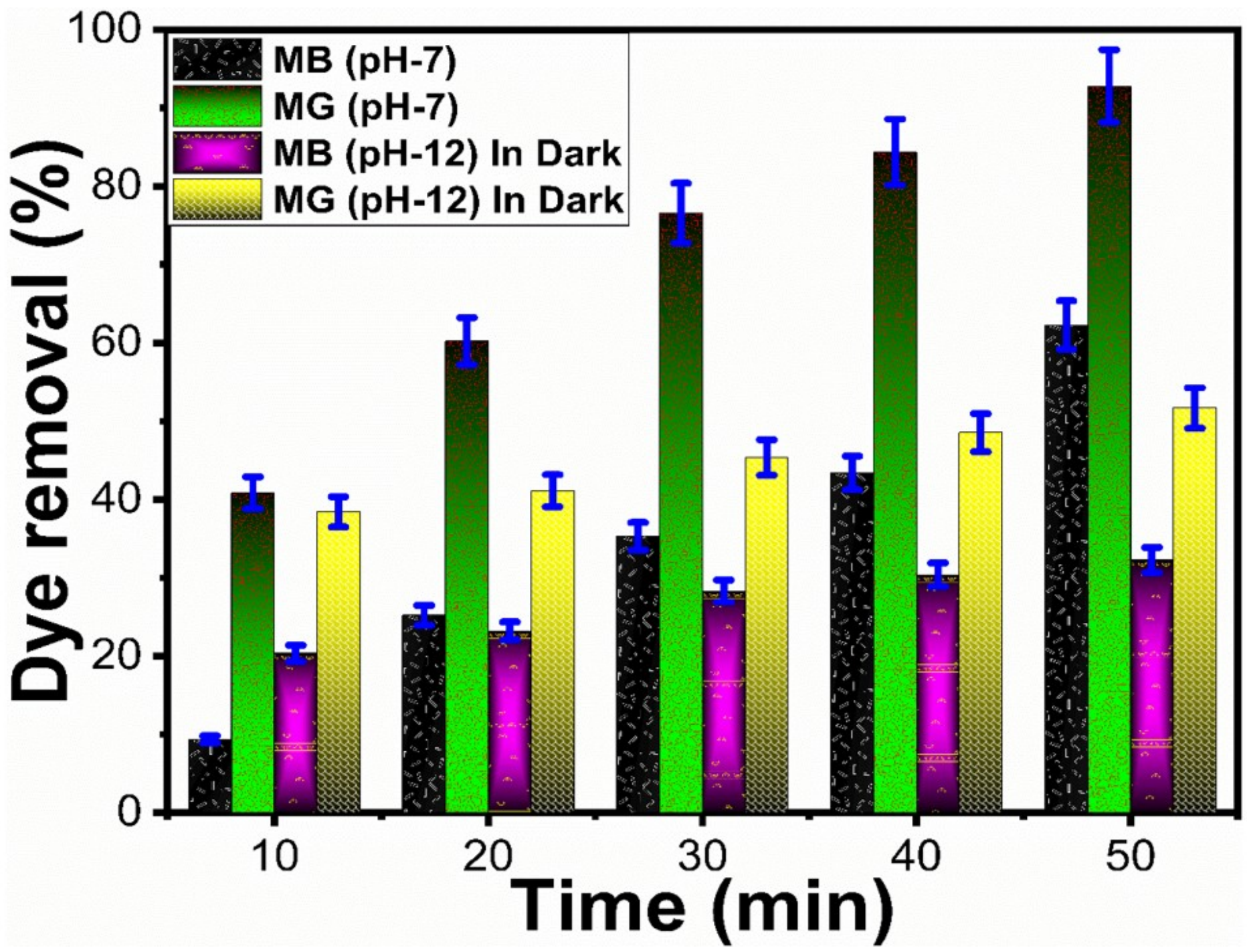

Figure (S4): Dye removal % under neutral pH 7 for MG dye 3.8×10^{-5}M and MB in 2.81 × 10⁻⁵M using 15mg photocatalyst dose of Datura leaves extract mediated ZnO (sample 4) under the illumination of sunlight. Also, the dye degradation using pH 12 for MG dye 3.8×10^{-5}M and MB in 2.81 × 10⁻⁵M in dark in the presence of 15mg photocatalyst dose of sample 4.

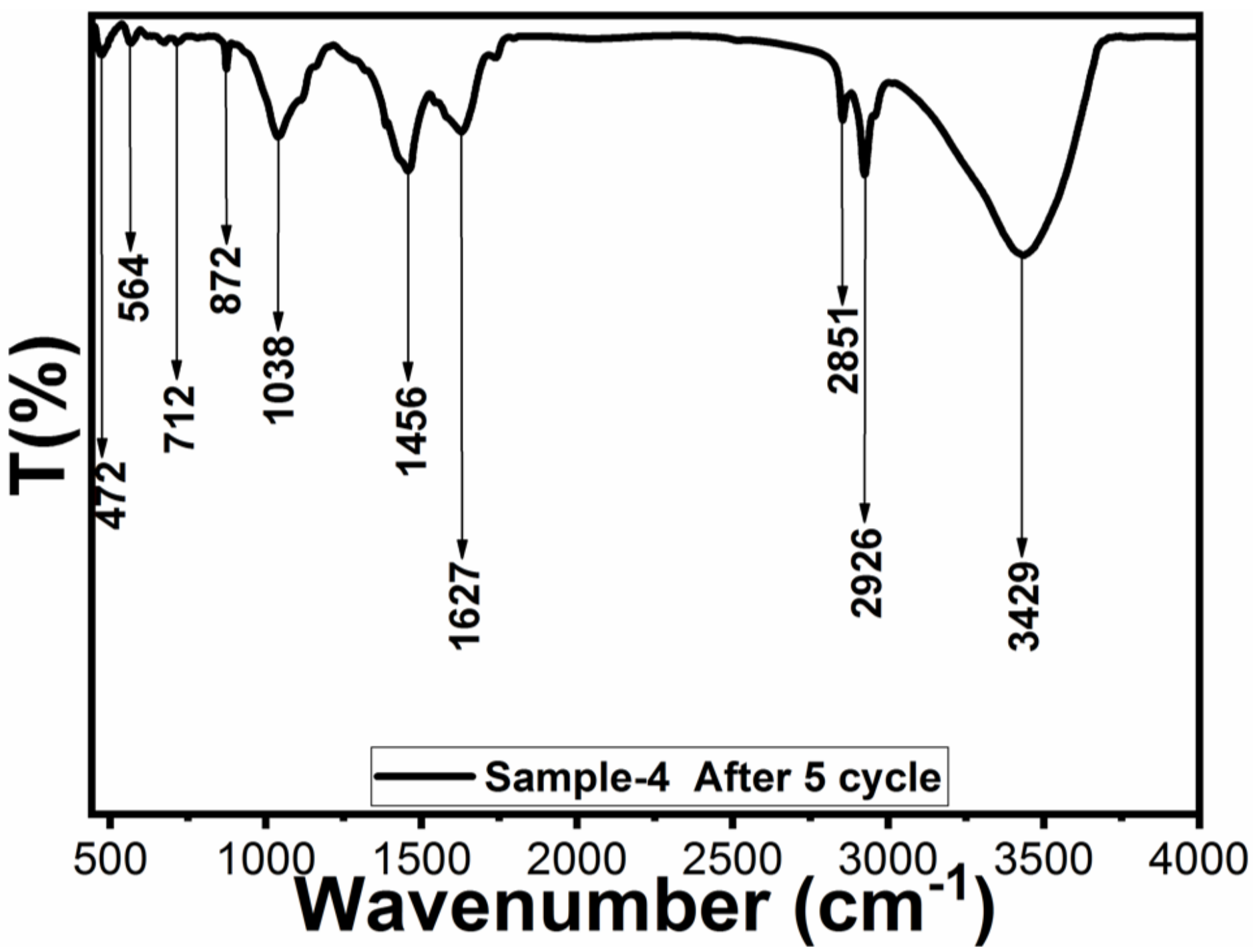

Figure (S5): FTIR analysis on the Datura leaves extract mediated ZnO (sample 4) after cycling test.

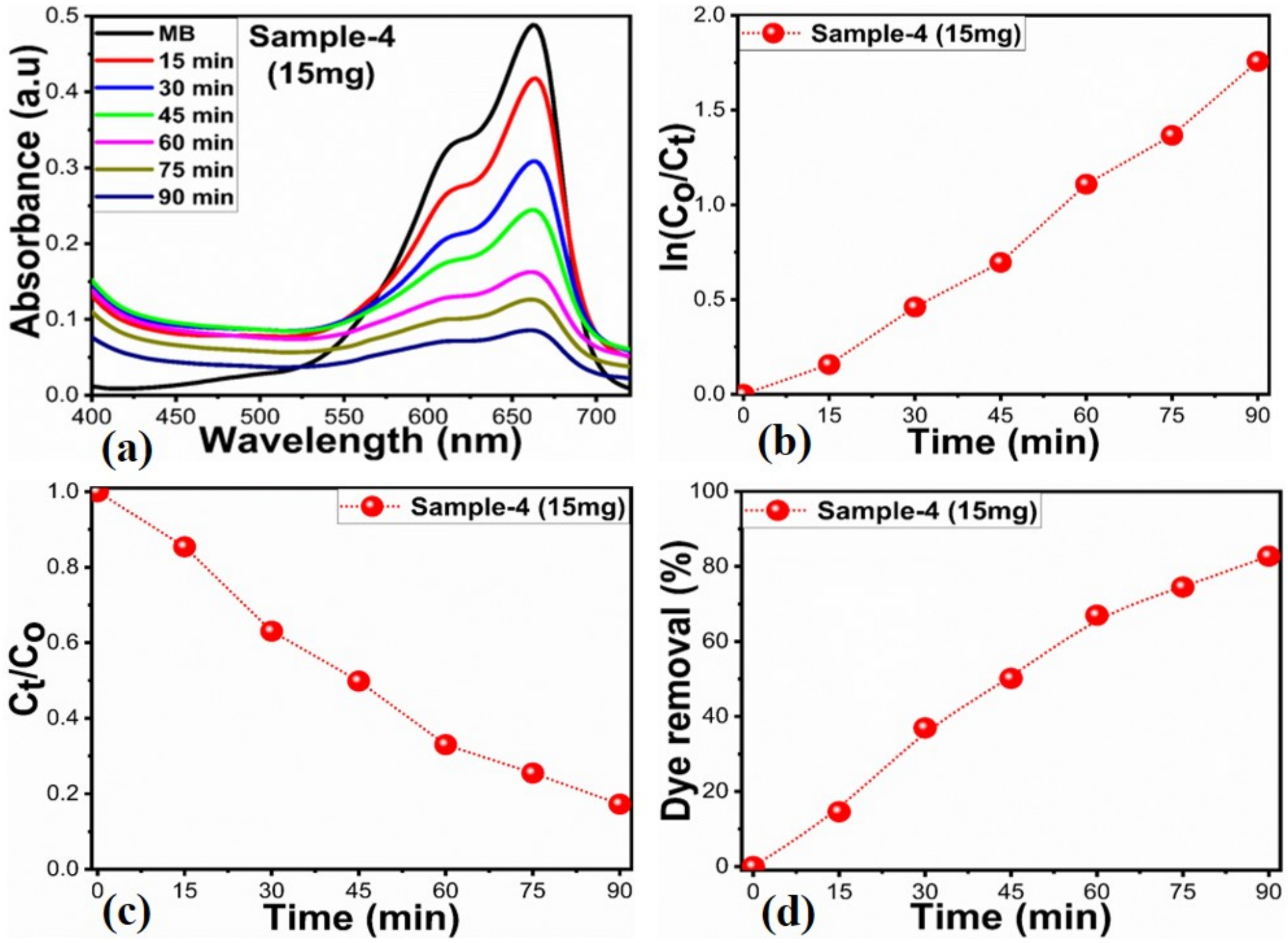

Figure (S6): (a) UV-visible absorbance spectra of MB in 2.81 × 10⁻⁵M in tape water using 15mg photocatalyst dose of Datura leaves extract mediated ZnO (sample 4), (b) pseudo first order kinetics, (c) dye degradation rate, (d) dye removal %

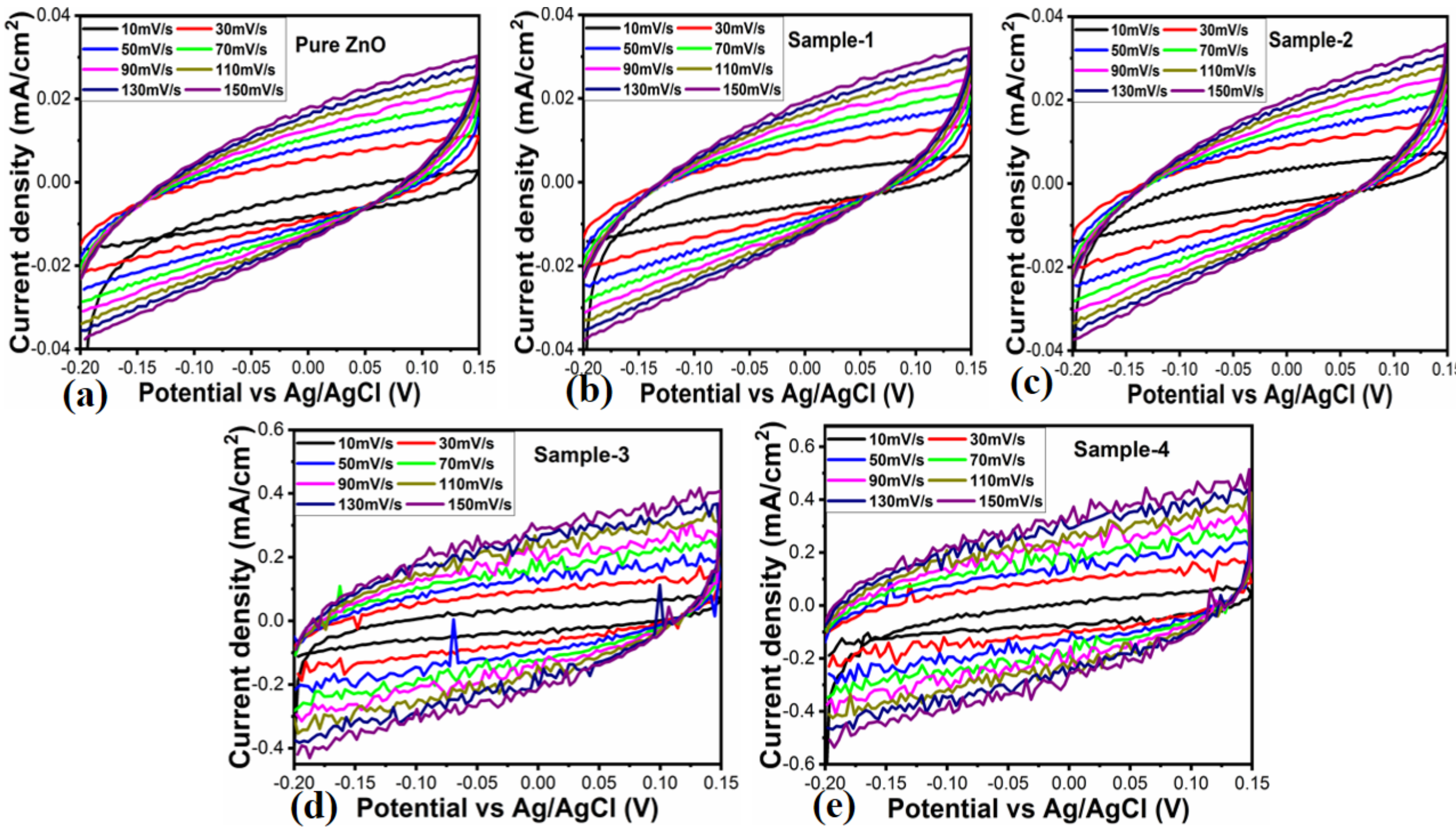

Figure (S7): Non-faradic CV cycles measured at different scan rates (a) pure ZnO, (b-e) sample 1 sample 2, sample 3, and sample 4

Table (S1): The estimated crystallite size of pure ZnO and Datura leaves extract mediated ZnO (sample 1-sample 4)

Sample (ID)	2 Theta (deg)	Peak position	FWHM	Height	Crystalline size (nm)
Pure ZnO	101	36.3375	0.32164	559	26.0
Sample-1	101	36.3125	0.16782	297	50.0
Sample-2	101	36.3125	0.38827	455	22.0
Sample-3	101	36.1375	0.41127	294	20.0
Sample-4	101	36.3625	0.22246	371	38.0

Table (S2): Comparison study of performance evaluation of as synthesized photocatalyst

**Catalyst**	**Synthesis method**	**Dye amount**	**MG removal (%)**	**Time (min)**	**Light Source**	**References**
ZnO NP	Green synthesized	20ppm	95	300	UV light	1

ZnO disc-like spherical NP	Precipitation method	21.6 mg/L	92	120 min	UV light	2
Mi-ZnO-NPs	Green synthesized	500 μl	49.27%	48 h	visible light	3
Ni/ZnO	wet-impregnation method	60 μg.ml^{-1}	99.4	40min	visible light	4
ZnO/SnO_2 -0.05	Bio-assisted synthesis	10 μg/mL	89.12%	80 min	visible light	5
ZnO–GO	Modified Hummer's method	10 ppm	98.51	20 min	UV light	6
GO-ZnO/Mn_2O_3	conventional solvothermal route	20 mg/L	65.85%	30 min	sunlight	7
CZnO–dots	single-pot and straightforward microwave process	0.5 g/L	94.8%	60 min	Visible light	8
MAl_2O_4@AC nanocomposites	In situ co-precipitation method	10×10^{-5}M	100	90	Visible light	9

$TiO_2/YMnO_3$	High-speed stirring technique	6.0×10^{-5}M	95.3	120V	Visible light	10
**Sample-4**	Green synthesized	3.8×10^{-5}M	**99.6**	60	**Sun light**	**Here in**

1. S.A. Devi, K.J. Singh and K.N. Devi, *Integrated Ferroelectrics*, 2020, ***205*** (1), 38-51.
2. P.L. Meena, K. Poswal and A.K. Surela, *Water and Environment Journal*, 2022, ***36***, 513-524.
3. M. Nasir, A. Naeem, L.Z. Khan, N. Zaib, H. Khan, Z. Najam and A. Gul, *Scientific Reports*. 2026.
4. S. Aziz, N.U. Rehman, H. Soonmin and N. Ahmad, *Civil Engineering Journal*, 2024, ***10***, 2601-2614.
5. B.A. Berehe, M.T. Yimer, S.L. Mekuria, G. Gedda, Y.J. Park, A.H. Assen, M.G. Pang and W.M. Girma, *Nano Select*, 2026, ***7***, e70121.
6. S. Stanković, M. Radovanović, S. Đorđievski, V. Nedelkovski, B. Markoli, S. Milić and M. Antonijević, *Metals*, 2026, ***16***, 71.
7. D.R. Rout, S. Chaurasia and H.M. Jena, *Journal of environmental management*, 2022, ***318***, *115449*.
8. A. Sekar and R. Yadav, *Optik*, 2021, ***242***, 167311.
9. M. Arunkumar, A.S. Nesaraj, C.E.F. Christy and C.J. Kennady, *Nanotechnology for Environmental Engineering*, 2023, ***8***, 643-654.
10. Y. Yulizar, I. Abdullah, R.M. Surya and N.L. Alifa, *Journal of Environmental Management*, 2023, ***342***, 118139.

Table (S3): Comparison study of performance evaluation of as synthesized photocatalyst

**Photocatalyst**	**Synthesis method**	**Pollutant**	**Removal (%)**	**References**
ZnO/KC NCs	Chemical reduction method	MR	90%	[1]
HC/ZnONC	Chemical method	MB	90% to 97%	[2]
AAK/ZnO nanocomposites	Wet chemical precipitation method	MB	98 %	[3]
ZnO(7.5%)/Bentonite	Impregnation method	SR- 3BL	70%	[4]
ZnO/MK30 nanocomposites	Ultrasonication technique	MB	95.76%	[5]
ZnO/sepiolite photolytic composites	Solgel method	MB	93.5%	[6]
Cu_2O/ZnO/Kaolinite-based composite	Co-precopitation method	MB	93%	[7]

Sample-4	Hydrothermal method	MB	88.4%	Here in
**Electrochemical detection**				
**Catalyst**	**Linera range**	**Limit of detection**		
MAG94	0.01–1.1 (μM)	3.9 (μM)		[8]
MPTMS	1–14 (μM)	0.4 (μM)		[9]
SALLE	0.63–219 (μM)	0.19 (μM)		[10]
Ag-rGO	3–319.85 (μg/L)	3 (μg/L)		[11]
	1–3000 (μM)	0.765 (μM)		[12]
**Sample-4**	**0.005mM to 6mM**	**0.005mM**		**Here in**

1. T. Gul, I. Khan, B. Ahmad, S. Ahmad, A.A. Alsaiari, M. Almehmadi, O. Abdulaziz, A. Alsharif, I. Khan and K. Saeed, K., *Heliyon*, , 2023, **9**, e16738.
2. N. Choudhary, V.K. Yadav, H. Ali, D. Ali, B.O. Almutairi, S. Cavalu and A. Patel, *Water*, 2013, ***15***, 1427.
3. M.F. Ehsan, H.R. Barai, M.M. Islam, M.A.B.H. Susan, S.W. Joo and M.S. Miran, *Materials Today Communications*, 2023, ***36***, 106563.
4. B. Boutra and M. Trari, *Water Science and Technology*, 2017, ***75***, 1211-1220.
5. T. Chellapandi, G. Madhumitha, S.M. Roopan, M. Elamathi, K. Leeladevi, E.R. Nagarajan, D. Vadivel and D. Dondi, *Optical Materials*, 2023, ***142***, 114099.
6. C. Li, H. Li, G. He, Z. Lei and W. Wu, *Advances in materials science and engineering, 2021,* **1**, 5357843.
7. P.A. Fufa, G.B. Feysia, N.S. Gultom, D.H. Kuo, X. Chen, D.M. Kabtamu and O.A. Zelekew, *Nanotechnology*, 2022, ***33***, 315601.
8. B.H. Vilsinski, P.R. Souza, A.C. de Oliveira, M. C´esar Filho, A.J. Valente, E. C. Muniz, O. Borges, A.P. Gerola, W. Caetano and A.F. Martins, Materials Today, Communications 2021, **26**, 101889.

9. I.K. Tonl´e, E. Ngameni, H.L. Tcheumi, V. Tchi´eda, C. Carteret and A. Walcarius, Talanta, 2008, **74**, 489–497.
10. R.S. Razmara, A. Daneshfar and R. Sahrai, J. Ind. Eng. Chem. 2011, **17**, 533–536.
11. P. Chettri, V. Vendamani, A. Tripathi, M.K. Singh, A.P. Pathak and A. Tiwari, Appl. Surf. Sci. 2017, **406**, 312–318.
12. M.L. Wen, Y.B. Zhao, X. Chen and C.Y. Wang, J. Pharm. Biomed. Anal. 1999, **18**, 957–961.
